# Recent advances in the chemistry of α-hydoxyphosphonates: synthesis, reactivity, biological applications and theoretical study: a review from 2010 to 2025

**DOI:** 10.1039/d5ra07967e

**Published:** 2026-04-16

**Authors:** Zineb Aouf, Abdeslem Bouzina, Djawhara Chohra, Rihab Benabbas, Yousra Ouafa Bouone, Rayene Sayad, Houria Bentoumi, Malika Ibrahim-Ouali, Nour-Eddine Aouf

**Affiliations:** a Laboratory of Applied Organic Chemistry, Bioorganic Chemistry Group, Sciences Faculty, Chemistry Department, Badji Mokhtar-Annaba University Box 12 23000 Annaba Algeria zineb.aouf@univ-annaba.dz; b Ensemble TPR 52 Av. Escadrille Normandie Niémen Marseille 13013 France

## Abstract

α-Hydroxyphosphonates are a critically important class of organophosphorus compounds with broad applications across diverse fields. This review paper surveys recent advancements from 2010 to 2025, focusing on their synthesis, reactivity, and biological activities. Key studies published prior to 2010 are also cited to provide essential background and to acknowledge foundational contributions to the field. Emphasis is placed on key synthetic methodologies such as the Pudovik and Abramov reactions, developments in asymmetric synthesis using chiral catalysts, and the utility of the α-hydroxyl group as a reactive functional handle. Special attention is given to environmentally friendly strategies including catalytic processes, ultrasonication and microwave-assisted synthesis. Several examples of the diverse biological properties of α-hydroxyphosphonates are also discussed. In addition, theoretical studies using DFT and molecular modeling are highlighted for their role in elucidating structure–activity relationships and supporting drug discovery. This review aims to be a valuable reference for researchers working in the field of organophosphorus chemistry.

## Abstract 2

A comprehensive review of α-hydroxyphosphonates covering synthesis, reactivity, biological activities and theoretical studies reported from 2010 to 2025, highlighting recent advances and future perspectives.

## Graphical abstract

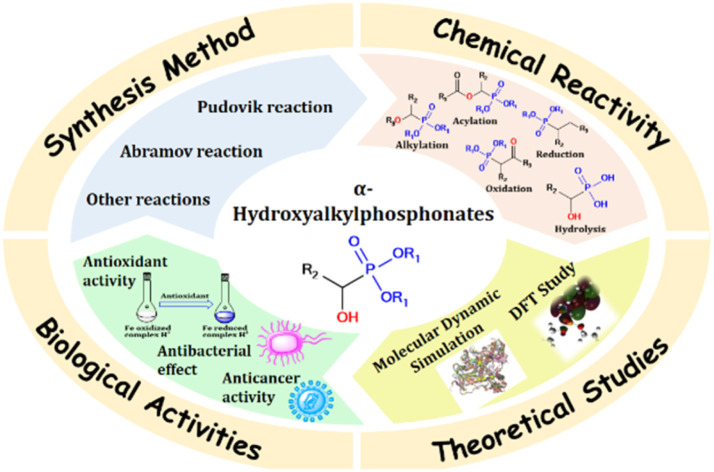

## 1. Introduction

Phosphonates are a diverse group of organic compounds that have attracted significant attention from scientists and researchers across various fields. These compounds are composed of phosphorus, carbon, oxygen, and hydrogen. They have found wide-ranging applications in industrial, agricultural, and medicinal chemistry due to their physical properties and their utility as synthetic intermediates.^1–4^ Specifically, α-hydroxyphosphonates constitute a significant category of phosphonates compounds, and their structure is found in various synthetic functional molecules.^5^

**Fig. 1:**
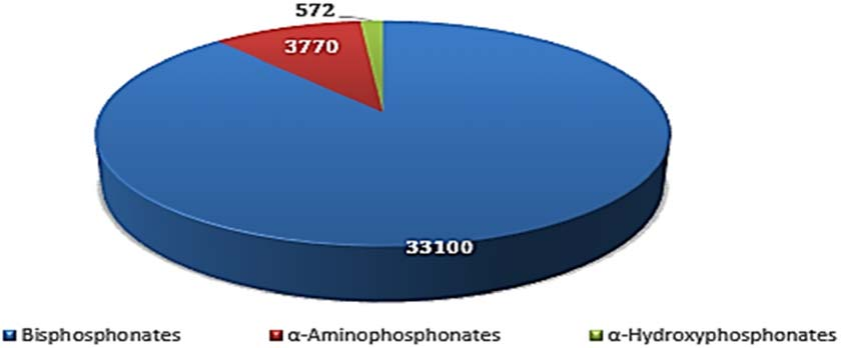
Bibliometric distribution of publications related to bisphosphonates, α-aminophosphonates and α-hydroxyphosphonates (Google Scholar, 2010–2025).

A bibliographic survey was conducted using the Google Scholar database with the keywords “bisphosphonates”, “α-aminophosphonates”, and “α-hydroxyphosphonates”. The search was performed in November 2025. The results indicated approximately 33 100 publications related to bisphosphonates, 3770 to α-aminophosphonates, and 572 to α-hydroxyphosphonates, showing that α-hydroxyphosphonates constitute the third most investigated family of phosphonate derivatives after bisphosphonates and α-aminophosphonates (Fig. 1).

The reported numbers correspond to the results returned by Google Scholar and should be considered as indicative bibliometric trends rather than absolute values, as the database may include different document types (research articles and reviews).

α-Hydroxyphosphonate derivatives display a large spectrum of therapeutic characteristics, including antiviral,^6^ enzyme inhibition,^7^ antibiotic,^8^ fungicidal,^9^ antivaccinia.^10^ Particularly, they also show significative antiproliferative activity against various human cancer cell lines,^11^ anti-HIV activity,^12^ and are potential anticancer agents.^13^ In addition to their diverse bioactive effects, α-hydroxyphosphonates have also proven to be versatile building blocks for accessing several structures that are more elaborate and potential bioactive compounds.^14–16^ This is expected due to their structural similarities with α-hydroxycarboxylic acids, which are known for their diverse biological activities.^17^

The importance of the chemistry of α-hydroxyphosphonate derivatives and their biological properties is illustrated by many reviews dedicated to this field in the last two decades.^18–24^

The Abramov^25^ and Pudovik^26^ reactions are two well-known methods for synthesizing α-hydroxyphosphonates. The Abramov reaction involves the hydroxylation process where dialkyl or triarylphosphites react with carbonyl compounds. On the other hand, the Pudovik reaction is based on adding “P-nucleophiles” (H-phosphonates) to an oxo compound.

**Fig. 2:**
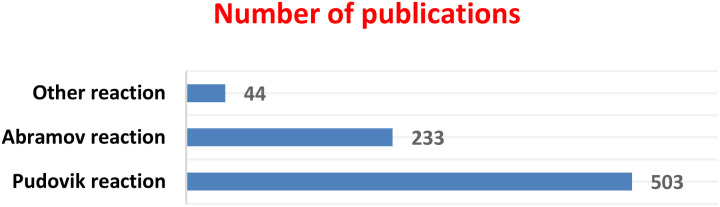
Bibliographic survey of synthetic methods for α-hydroxyphosphonates (2010–2025, Google Scholar data).

Since 2010, the Pudovik reaction has been the most widely used method for the preparation of α-hydroxyphosphonates, accounting for a large proportion of the reported publications (503 articles). In comparison, the Abramov reaction has been described in 233 publications, whereas other synthetic approaches represent a smaller fraction (44 articles), as illustrated in Fig. 2. These data were obtained from Google Scholar searches (accessed in November 2025) using the keywords “α-hydroxyphosphonate synthesis”, “hydroxyphosphonate Pudovik reaction”, “hydroxyphosphonate Abramov reaction”, and “α-hydroxyphosphonate synthesis other methods”. The reported numbers reflect the search results provided by the database and are intended to illustrate general research trends rather than exact bibliometric values.

In addition to these classical methods, the development of asymmetric synthesis of α-hydroxyphosphonates has gained increasing attention due to their broad biological and pharmacological applications. Various strategies have been explored, including the use of chiral catalysts, organocatalysts, and metal-based catalysts, as well as enantioselective Abramov reactions.^27^ These approaches enable the efficient production of enantioenriched α-hydroxyphosphonates, which are crucial for designing bioactive compounds and advanced functional materials.

The free hydroxyl group at the α-position of α-hydroxyphosphonates makes them highly versatile building blocks. This functional group allows for easy modification, leading to the creation of new families of compounds with significant applications in both synthetic chemistry and biology. Examples of these functionalized derivatives include α-amino, α-halo, α-keto, α-diketo, and α-acetoxy phosphonates.^28^

Theoretical studies of α-hydroxyphosphonates, using DFT calculations and molecular modeling, provide insights into their reactivity, stability, and stereoselectivity. These approaches help elucidate reaction mechanisms, electronic properties, and transition states, aiding in the design of bioactive compounds. Additionally, molecular docking predicts their interactions with biological targets, supporting drug discovery.

This review encompasses the period from 2010 to 2025, focusing on recent advances in the biological activities, synthesis, reactivity and some theoretical study of α-hydroxyphosphonates. While the primary focus is on the latest developments, we recognize the importance of foundational research. Therefore, relevant literature published prior to 2010 may also be included for a comprehensive understanding.

## 2. Medicinal applications of α-hydroxyphosphonates

α-Hydroxyphosphonates and their derivatives have emerged as a promising class of compounds in medicinal chemistry due to their diverse therapeutic potential and pharmacological properties. This growing field has seen significant advancements in the past decade.

A brief overview of the biological significance of α-hydroxyphosphonates, including illustrative examples, is outlined below.

### 2.1. Antibacterial activity of α-hydroxyphosphonates

**Fig. 3:**
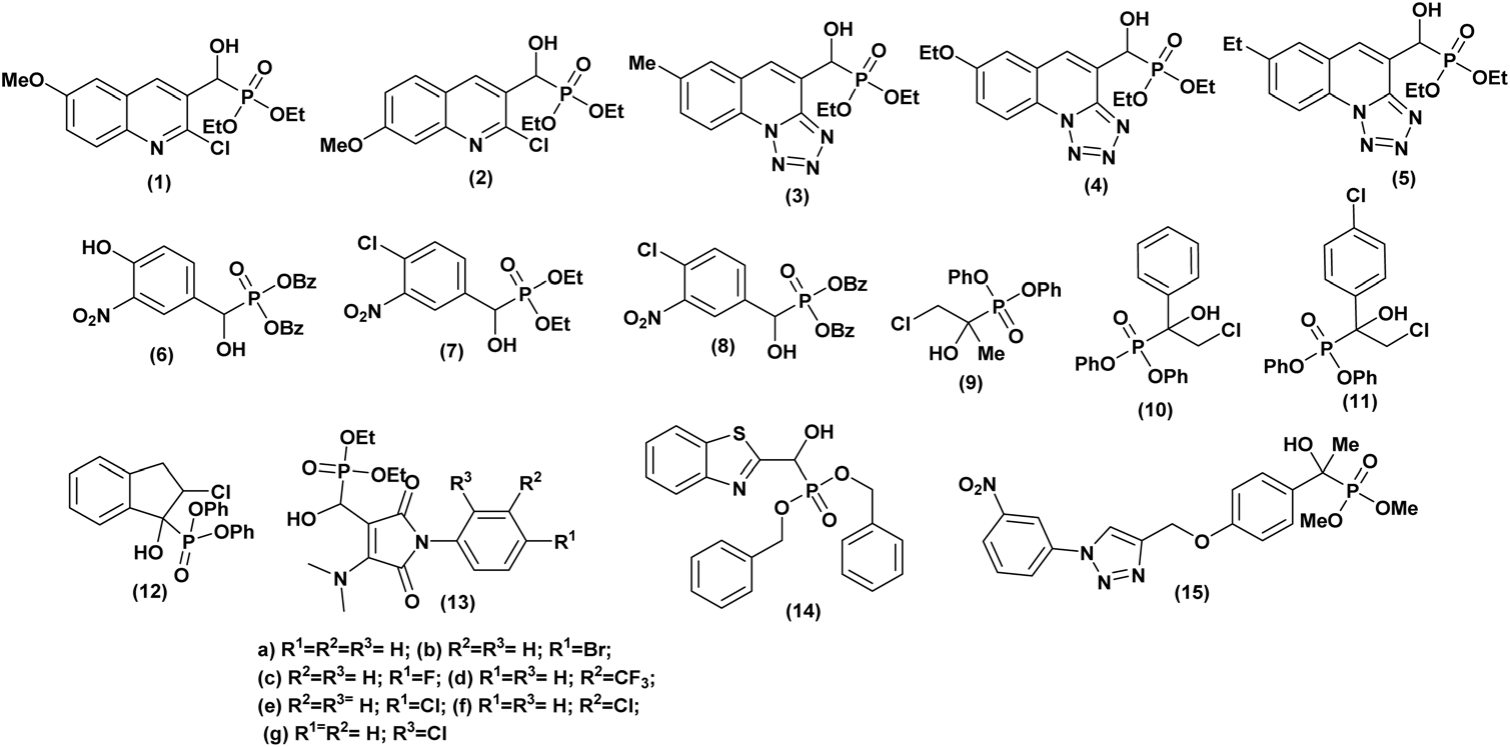
Chemical structures of antibacterially active α-hydroxyphosphonate derivatives.

An Indian team^29^ has demonstrated the *in vitro* antibacterial activity of some α-hydroxyphosphonate molecules derived from 2-chloroquinoline. These molecules were tested against both Gram-positive bacteria (*Staphylococcus aureus* and *Bacillus megaterium-I*) and Gram-negative bacteria (*Escherichia coli*, *Salmonella typhi*, and *Proteus vulgaris*). The results showed that compounds (1 and 2, Fig. 3) exhibited the best antibacterial activity. These compounds displayed activity comparable to the reference antibiotic, streptomycin.

Kategaonkar and co-workers^30^ synthesized a series of novel α-hydroxyphosphonate molecules incorporating tetrazolo[1,5-*a*]quinoline derivatives. These compounds underwent evaluation for potential antibacterial efficacy against both Gram-positive (*Bacillus subtilis*) and Gram-negative (*Escherichia coli*) bacterial strains, with streptomycin employed as a reference compound. The majority of the tested compounds demonstrated significant antibacterial activity. Especially, compounds 3, 4 and 5 (Fig. 3) exhibited pronounced activity against *B. subtilis*, while compounds 3, and 5 displayed notable efficacy against *E. coli*, closely resembling the effectiveness of the standard antibiotic utilized.

Another study was conducted by Subba Reddy *et al.*,^31^ wherein α-hydroxyphosphonates compounds were assessed for their antimicrobial activity against *Staphylococcus aureus* (ATCC 25923) (Gram-positive) and *Escherichia coli* (ATCC 25922) (Gram-negative) using the disc diffusion method at varying concentrations (50, 100 ppm). Compounds 6, 7 and 8 (Fig. 3) exhibited the highest efficacy, while the others demonstrated moderate antibacterial activity against both *Staphylococcus aureus* and *Escherichia coli*, in comparison to Penicillin, which served as the reference drug. Phillips *et al.*,^32^ investigated the potential of α-hydroxyphosphonates as antimicrobial agents. They synthesized two series of these compounds, differentiated by their ester protecting groups: cyclic phosphonate esters and phenyl phosphonate esters. The researchers tested their *in vitro* activity against various bacterial strains, including both Gram-negative (*Escherichia coli*, *Acinetobacter haemolyticus*, *Stenotrophomonas maltophilia*, *Pseudomonas aeruginosa*) and Gram-positive (*Bacillus cereus*, *Staphylococcus aureus*). The study revealed that α-hydroxyphosphonate derivatives 9, 10, 11, and 12 (Fig. 3) all sharing a diphenyl ester protecting group, exhibited the most potent antibacterial activity. These compounds inhibited the growth of both Gram-negative and Gram-positive bacteria more effectively than others. Interestingly, compound 11 demonstrated comparable or superior bactericidal activity against several Gram-negative strains compared to standard antibiotics like chloramphenicol. In most cases, they also outperformed fosfomycin. Another work published in 2014 by Patil and co-workers^33^ reported the synthesis of a series of novel compounds containing α-hydroxyphosphonate moiety. These compounds were evaluated for both qualitative and quantitative antimicrobial activities against various pathogenic bacteria and fungi.

The tested organisms included Gram-positive bacteria (*Bacillus subtilis*, NCIM 2250), Gram-negative bacteria (*Escherichia coli*, ATCC 25922), and fungi *like Candida albicans* (MTCC 277), *Candida tropicalis* (MTCC 184), *Aspergillus Niger* (MCIM 545), and *Aspergillus clavatus* (MTCC 132). The obtained results revealed that among the tested molecules, the phosphonates (13a–g, Fig. 3) exhibited moderate to good antibacterial activity compared to reference antibiotics, with activity notably enhanced by the presence of pharmacologically active substituents (F, Cl, Br, CF_3_) on the aromatic ring, regardless of their position in the molecule. Conversely, compounds lacking these substituents displayed the least antibacterial effects.

Furthermore, Yeswanth *et al.*,^34^ evaluated the antimicrobial activity of a novel α-hydroxyphosphonate derivative, dibenzyl (benzo[*d*]thiazol-2-yl(hydroxy)methyl) phosphonate (14), against penicillin, ampicillin, and methicillin resistant strains of *Staphylococcus aureus* (MRSA). The study showed significant results, with (compound 14, Fig. 3) inhibiting the planktonic biofilms associated with the rapid spread of persistent MRSA infections. This was revealed by dot blot and zymogram analysis. Additionally, the concentration of compound 14 appeared to correlate with increased lysis (breakdown) of bacterial biofilms and aggregates.

The last example, described by Telu *et al.*,^35^ synthesized a library of novel 1,2,3-triazolo phosphonate compounds and evaluated their antibacterial activity against various Gram-positive (*Staphylococcus aureus*) and Gram-negative (*Escherichia coli*, *Klebsiella pneumoniae*, and *Pseudomonas aeruginosa*) bacterial strains. Their study found that most of the compounds exhibited good to moderate activity in inhibiting the growth of the tested species.

Particularly, dimethyl (1-hydroxy-1-(4-((1-(3-nitrophenyl)-1*H*-1,2,3-triazol-4-yl)methoxy)phenyl)ethyl) phosphonate (compound 15, Fig. 3) displayed good antibacterial effects against all the strains. The study also suggests that the presence of the α-hydroxyphosphonate moiety at the para-position of the aryl ring may be favorable for antibacterial activity. Additionally, researcher used *in silico* docking to predict that molecule (15) exhibits strong interactions with the DNA active site, potentially explaining its broad-spectrum activity.

### 2.2. Anticancer activity of α-hydroxyphosphonates

**Fig. 4:**
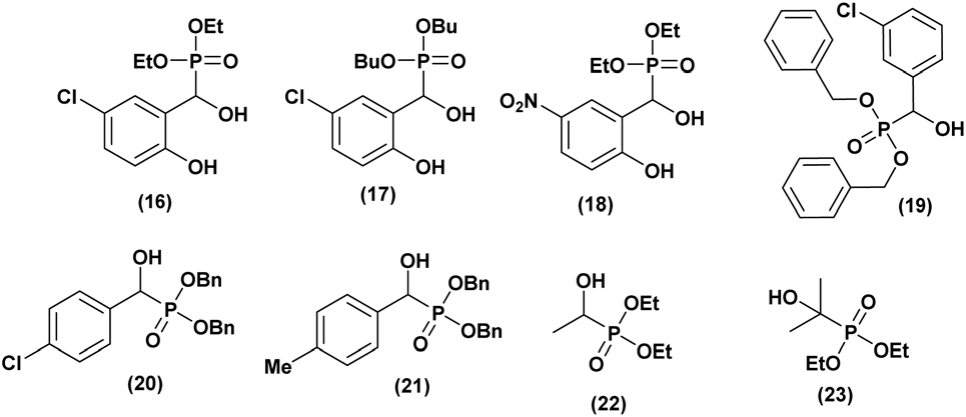
Chemical structures of anticancer active α-hydroxyphosphonate derivatives.

Kalla *et al.*^36^ synthesized a series of α-hydroxyphosphonates and evaluated their cytotoxic activity against two cancer cell lines (the human alveolar basal epithelial adenocarcinoma cell line (A549) and the epidermal cancer cell line (KB)). Using the MTT assay to assess growth inhibition, they found that all compounds exhibited dose-dependent cytotoxic effects against both cell lines. Among them (compounds 16, 17, and 18, Fig. 4), demonstrated significant cytotoxicity. Notably, compound 16 effectively inhibited A549 cell proliferation at a concentration of 2 µM, making it a promising candidate for cancer treatment. Additionally, their structure–activity relationship (SAR) analysis revealed that nitro- and chloro-substituted compounds exhibited strong activity, which was further enhanced by ethoxy and butoxy substitutions on the phosphorus atom.

In another study, Ràdai and co-workers^37^ synthesized a series of α-hydroxyphosphonates and evaluated their anticancer activity against the Mes-SA human uterine sarcoma cell line. Additionally, the IC_50_ values of the most effective compounds were determined. All screened dibenzyl α-hydroxyphosphonates exhibited high cytotoxic potency. Furthermore, compounds with *ortho*- and *meta*-chloro or methyl substitutions in the benzene moiety demonstrated greater toxicity compared to their *para*-substituted analogues. Among them, dibenzyl 1-(3-chlorophenyl)-1-hydroxymethyl phosphonate (19, Fig. 4), featuring a *para*-chloro substitution, was identified as the most promising cytotoxic agent, exhibiting a lower IC_50_ than three standard chemotherapeutic drugs: doxorubicin, carboplatin, and dacarbazine, commonly used to treat uterine sarcoma.

The same research group^38^ investigated the cytotoxic effects of α-hydroxyphosphonate, against Mes-SA uterine sarcoma cells and the multidrug-resistant (MDR) cell line Mes-SA/Dx5. Additionally, a structure–activity relationship (SAR) study was conducted to assess the impact of substitution variations on the benzene moiety to enhance cytotoxic activity.

Cytotoxicity screening revealed that all tested compounds were toxic to both cell lines. Notably, compound (20) exhibited selective toxicity toward Mes-SA/Dx5. Further evaluations, including fluorescence protein-based assays, were performed to determine the IC_50_ values of these compounds. Compounds with *para*-chloro (20) and *para*-methyl (21) (Fig. 4) substitutions were found to be less toxic against Mes-SA mCh cells compared to their analogues, which exhibited low IC_50_ values ranging from 83 to 105 µM. Moreover, the other compounds demonstrated toxicity against Mes-SA/Dx5 mCh cells, with IC_50_ values ranging from 35 to 221 µM. Interestingly, the SAR study confirmed that substitutions at the *meta* position (chlorine, NO_2_, and methyl) on the benzene ring significantly enhanced cytotoxic potency.

A Hungarian research^39^ group recently synthesized a series of α-hydroxy-alkylphosphonates and α-hydroxy-alkylphosphine oxides. The cytotoxic activity of these compounds was evaluated by assessing the cell viability of U266 myeloma and PANC-1 pancreatic adenocarcinoma cell lines at concentrations of 1, 10, and 100 µM. The viability assays indicated that most compounds exhibited minimal toxicity, maintaining viability close to 1.0 across all concentrations. However, for U266 cells, greater variability was observed, particularly at 100 µM, where phosphonates 22 and 23 (Fig. 4), as well as phosphine oxide 23, demonstrated moderate toxicity, reducing cell viability to 0.77–0.95. Among them, hydroxyphosphonate 23 exhibited the highest toxicity, inducing 23% cell death.

### 2.3. Antioxidant activity of α-hydroxyphosphonates

Numerous studies have highlighted the significant role of free radicals in the development of various diseases, including cancer, immune system degeneration, cardiovascular diseases, and inflammatory disorders. Free radicals are highly reactive chemical species generated during metabolic processes, which can induce the oxidation of biomolecules such as DNA, lipids, proteins, and carbohydrates, potentially causing damage to living cells. To counteract these harmful effects, the body relies on a defense system composed of enzymatic and non-enzymatic antioxidants, which help neutralize free radicals and maintain optimal health.

**Fig. 5:**
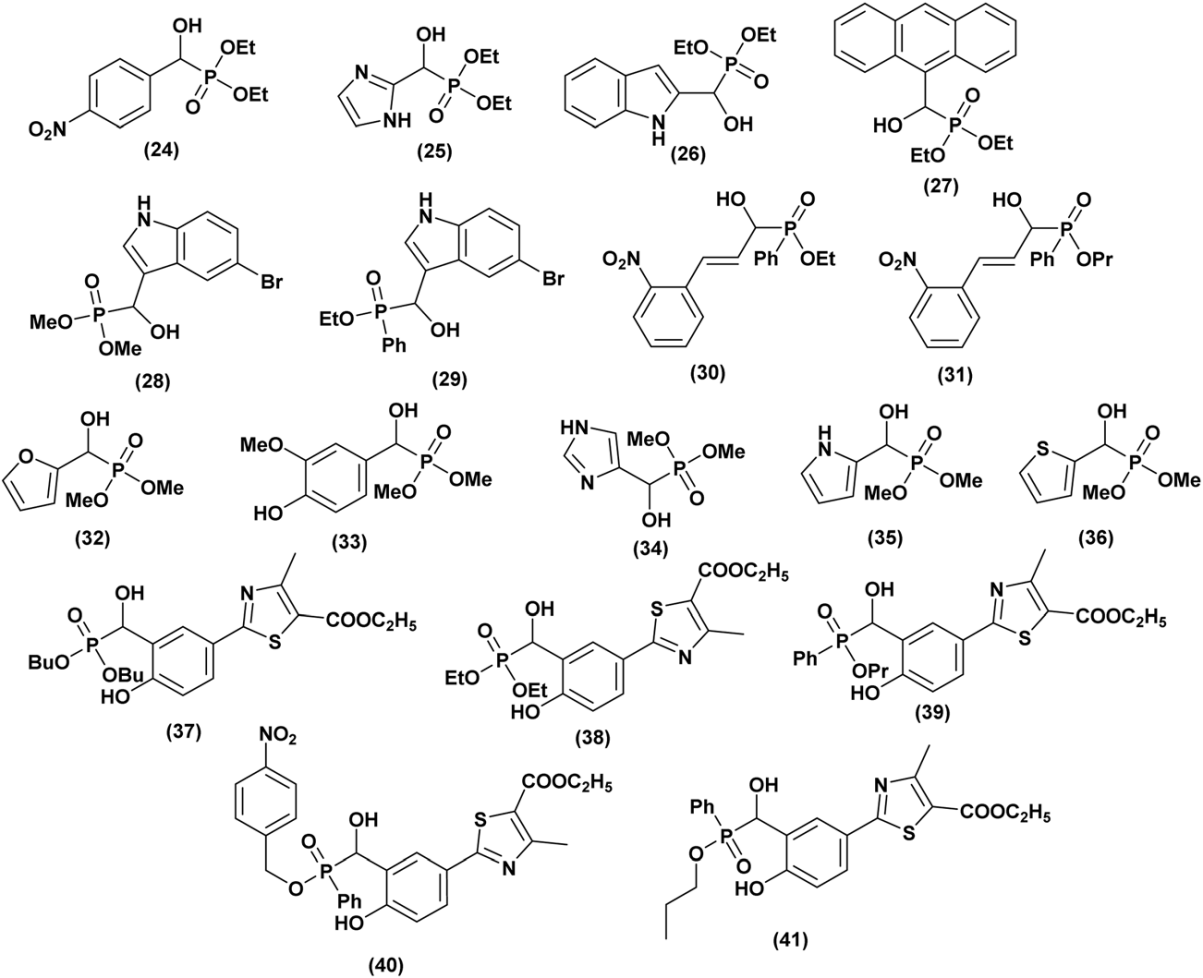
Chemical structures of antioxidant active α-hydroxyphosphonate derivatives.

In this context, Rao *et al.*^40^ synthesized a series of ten α-hydroxyphosphonate compounds and evaluated their antioxidant activity using the DPPH test, reducing power assay, and lipid peroxidation assay. The authors reported that three compounds 24, 25, and 26 (Fig. 5), exhibited potent antioxidant activity. Among them, compound (26) demonstrated the highest reducing power, while compound (24) showed the strongest DPPH scavenging activity and hydroxyl radical scavenging activity. This enhanced activity is attributed to the presence of a –NO_2_ group, a highly electron-withdrawing moiety, which influences electron and hydrogen-donating capacities. By decreasing the electron density of the P=O group, it increases the compound's affinity for free radicals, thereby enhancing its antioxidant potential.

In 2012, Naidiu *et al.*^41^ successfully synthesized a series of α-hydroxyphosphonates using the Abramov reaction and evaluated their antioxidant potential through lipid peroxidation (LPO), reduced glutathione (GSH), superoxide dismutase (SOD), and total antioxidant capacity (TAC) assays. When comparing the antioxidant activities of these compounds to vitamin C, the authors found that all exhibited significant antioxidant capacity. However, diethyl anthracen-9-yl (hydroxy) methylphosphonate (27, Fig. 5) demonstrated the highest activity against free radicals, followed by compounds containing electron-withdrawing groups (2-Cl, 4-Cl, 3-NO_2_, 2-NO_2_, 4-NO_2_). In contrast, their electron-donating analogues (4-OMe, 3,4-Di-OMe, 4-CH_3_, 4-*i*Pr, 4-N(CH_3_)_2_) displayed only moderate antioxidant activity. Rasheed and co-authors,^42^ evaluated the *in vitro* and *in vivo* antioxidant activity of five α-hydroxyphosphonates synthesized under ultrasonic conditions. To minimize animal mortality, an *in vitro* study was conducted as a preliminary screening method. The non-enzymatic antioxidant activity was assessed using DPPH and superoxide radical scavenging assays, revealing that all compounds exhibited moderate to potent antioxidant activity. Among them, compounds (28, 29, 30, and 31, Fig. 5) demonstrated the highest activity in both methods, prompting further *in vivo* evaluation. The catalase (CAT) assay confirmed the strong antioxidant potential of these four compounds in enzymatic tests as well. Especially, compound (31) exhibited the highest free radical scavenging capacity at both tested concentrations, with an IC_50_ value comparable to ascorbic acid, making it the most effective among the tested compounds. Variations in scavenging activity were attributed to differences in substituents at the α-carbon, influencing reactivity toward free radicals.

In 2014, Santhisudha and co-workers^43^ developed a green and efficient synthesis of a series of α-hydroxyphosphonates and evaluated their *in vitro* antioxidant activity using multiple assays, including DPPH, hydrogen peroxide scavenging, nitric oxide scavenging, and FRAP, with ascorbic acid as the reference. Compounds 32, 33, 34, 35, and 36 (Fig. 5) exhibited the highest antioxidant capacity in both nitric oxide and hydrogen peroxide scavenging assays. In the FRAP assay, compounds 32, 33, 34, and 35 showed the strongest activity, while in the DPPH test, 32, 33, and 34 demonstrated important antioxidant potential. The structure–activity relationship study revealed that OH and NH substitutions significantly enhance antioxidant activity.

In the last, Sampath *et al.*^44^ reported the novel synthesis of α-hydroxyphosphonates and evaluated their antioxidant potential by examining their ability to scavenge DPPH free radicals. All tested compounds exhibited promising antioxidant activity. The antioxidant activity was assessed at concentrations of 50, 100, 200, 400, and 600 µg mL^−1^. The results indicated that compounds (37, 38, 39, 40, and 41, Fig. 5) demonstrated the highest potency, with 37 and 39 showing the strongest effects. The remaining compounds exhibited moderate antioxidant activity, with ascorbic acid used as the standard reference.

Additionally, the synthesized compounds exhibited varying antibacterial activity against Gram-positive and Gram-negative bacteria and prominent antifungal effects, with 38, 39, 40, and 41 being the most effective. Streptomycin and Bovastin were used as reference drugs, confirming their promising antimicrobial potential.

### 2.4. Anti-norovirus activity

**Fig. 6:**
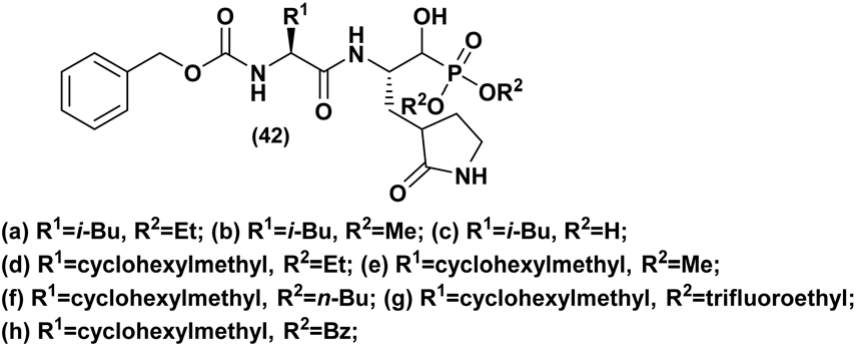
Chemical structures of anti-norovirus active α-hydroxyphosphonate derivatives.

Norovirus is a single-stranded RNA virus from the Caliciviridae family. It is a nonenveloped virus, lacking an outer lipid membrane. Norovirus is recognized for inducing gastroenteritis, resulting in symptoms like diarrhea, vomiting, stomach cramps, and nausea. In this context, an American team^45^ synthesized a series of dipeptidyl α-hydroxyphosphonates and investigated their effectiveness against norovirus by targeting the inhibition of 3C-like cysteine protease (3CLpro) in a cell-based replicon system. Additionally, they assessed the enzyme selectivity of these compounds using a panel of proteases. The results clearly demonstrated the strong inhibitory effects of the α-hydroxyphosphonate derivatives. Furthermore, substituting P2 leucine with cyclohexyl alanine was found to enhance the anti-norovirus activity by improving cellular permeability, as evidenced by the comparison between (42a/42d and 42b/42e, Fig. 6). Moreover, altering the substituent on R2 significantly influenced the potency of the activity, particularly with compounds 42d and 42g, which exhibited the highest efficacy.

### 2.5. Herbicidal properties

**Fig. 7:**
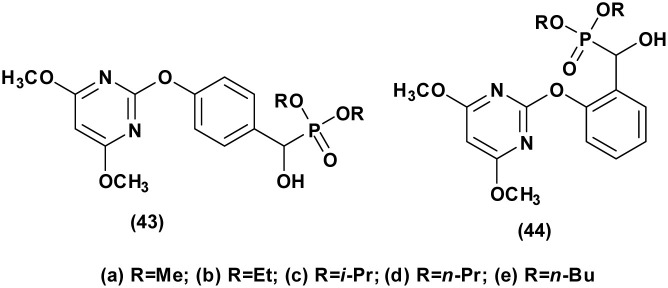
Chemical structures of herbicidal active α-hydroxyphosphonate derivatives.

Herbicides function by disrupting normal plant growth and development processes through various biological mechanisms or enzymes, effectively controlling susceptible plants. In a study conducted by Song *et al.*,^46^ the efficacy of α-hydroxyphosphonate derivatives as herbicides was underscored. Their research explored the herbicidal potential of compounds (43 and 44, Fig. 7) against *Brassica campestris* L and *Echinochloa crus-galli*, revealing a greater efficacy against *Brassica campestris* L in most instances. Moreover, the study demonstrated that these compounds exhibited notable and selective herbicidal effects against amaranth pigweed (*A. retroflexus*) in both pre- and post-emergence treatments, with some compounds achieving complete inhibition during pre-emergence treatment and significant inhibition rates during post-emergence treatment. For instance, compounds 43a, 43b, and 43e demonstrated complete inhibition of *A. retroflexus* during pre-emergence treatment, and inhibition rates of 91.7%, 98.8%, and 99.5% during post-emergence treatment at a dosage of 1.5 kg ha^−1^, respectively.

To further rationalize the observed biological activities, representative computational studies, including molecular docking and ADME analyses, have also been explored.

### 2.6. *In silico* studies supporting biological activities

**Fig. 8:**
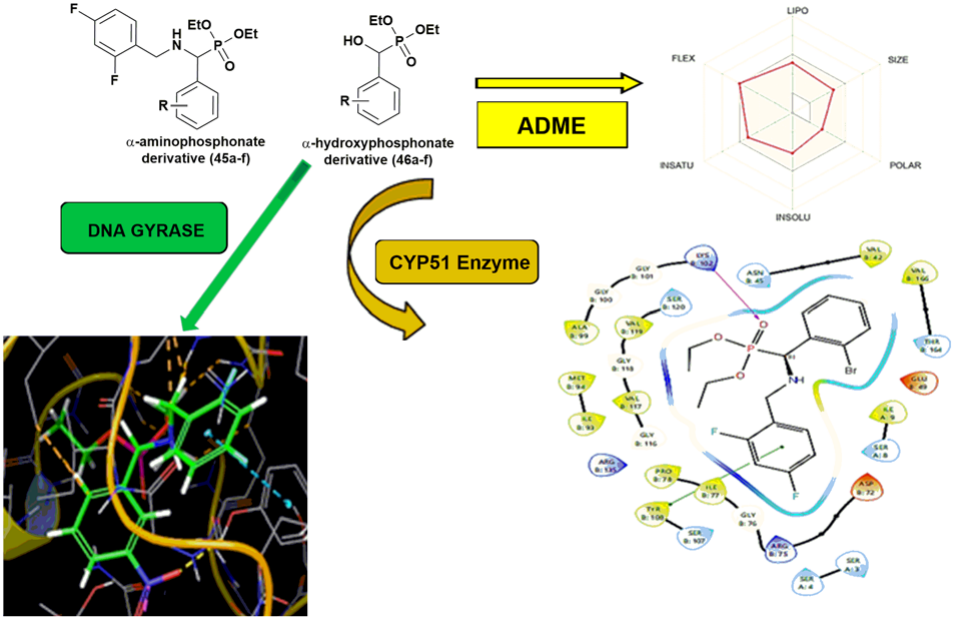
Computational ADME and molecular docking studies of α-aminophosphonate and α-hydroxyphosphonate derivatives.

To rationalize the observed biological activity of α-hydroxyphosphonate derivatives, molecular docking studies were performed against DNA Gyrase B and CYP51 enzymes. In this context, Ummadi *et al.*^47^ evaluated α-aminophosphonate (45a–f) and *α*-hydroxyphosphonate derivatives (46a–f), revealing favorable pharmacokinetic properties and notable inhibitory activity. The core structures of the synthesized derivatives and the computational analyses are summarized in Fig. 8.

ADME analyses indicated that all compounds satisfied Lipinski's rule, suggesting good oral bioavailability comparable to Ciprofloxacin and Fluconazole. All compounds showed high gastrointestinal absorption, good skin permeability, and no PAINS alerts. BOILED-Egg predictions suggested that several derivatives could cross the blood–brain barrier, and most were not substrates of P-glycoprotein. CYP inhibition profiling indicated potential inhibition of multiple cytochrome P450 isoenzymes.

Molecular docking revealed stronger binding affinities of the synthesized compounds toward DNA Gyrase B and CYP51 compared with reference drugs, mainly stabilized through hydrogen bonding, π–π interactions, and hydrophobic contacts. Notably, α-aminophosphonate derivatives exhibited slightly better interactions than the corresponding α-hydroxyphosphonate analogues. Overall, these *in silico* studies highlight the promising pharmacokinetic profiles and binding affinities of α-aminophosphonate and α-hydroxyphosphonate derivatives, providing insights into structure–activity relationships and supporting their potential as antimicrobial agents.

## 3. Synthetic approaches of α-hydroxyphosphonates

### 3.1. *Via* abramov reaction conditions

#### 3.1.1. General mechanism and reaction features

**Fig. 9:**
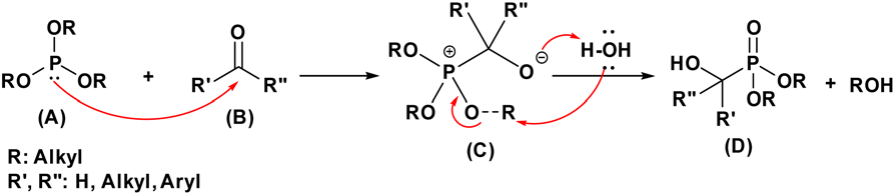
Mechanistic proposal for the Abramov reaction.

The Abramov reaction is an important chemical reaction in organic chemistry, named after the Russian chemist Vladimir Abramov, who first reported it in 1950.^25^ Mirroring the nucleophilic addition to carbonyls, the plausible mechanism of the Abramov reaction begins with the phosphorus atom in the trialkyl phosphite (A) attacking the electrophilic carbonyl carbon (B). This leads to the formation of a tetrahedral intermediate (C), characterized by newly formed C–O and P–O–C bonds. Traditionally, an alkyl group shifts from a phosphorus-bound oxygen to the emerging alkoxide. However, under aqueous conditions,^48^ this alkyl transfer is likely bypassed in favor of a direct hydroxide attack or protonation by water. Rather than an internal proton shifts to an alkoxy group, water donates a proton directly to the negatively charged oxygen of the intermediate. This protonation, accompanied by the formation of the stable P=O bond, yields the α-hydroxyphosphonate (C), with an alcohol (ROH) eliminated as a byproduct (Fig. 9).

In the following section, we provide several examples of carbonyl phosphorylation *via* the Abramov reaction. We examine the impact of various reaction conditions and catalytic systems on the efficiency and selectivity of the process, including the specific challenges associated with asymmetric induction. For clarity and better visualization, the key findings across these different methodologies are summarized in Table 1.

**Table 1:** Some examples of Abramov reaction for α-hydroxyphosphonates

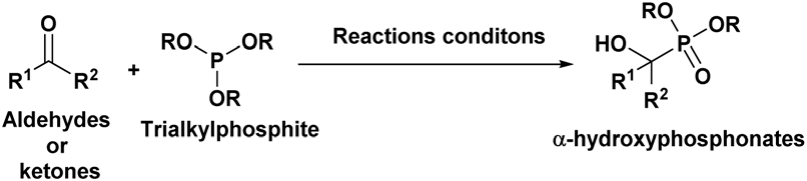					
Entry	Aldehydes or ketones	Trialkylphosphite	Reaction conditions	Yield %/Time (min)	Ref.
1	R^1^: H/R^2^: 4-ClC_6_H_4_, 4-BrC_6_H_4_, 4-CO_2_MeC_6_H_4_, 4-CF_3_C_6_H_4_, 1,4-di-OMe-2-NO_2_C_6_H_2_, 1,3-di-ClC_6_H_3_, naphthal, 3a^1^,5a^1^-dihydropyrene	R: Et	ZnBr_2_ (10 mol%), solvent free, rt	79–91%, 10–30 min	49
2	R^1^: H/R^2^: C_6_H_5_, 4-OHC_6_H_4_, 4-ClC_6_H_4_, C_6_H_5_CH = CH, 4-NO_2_C_6_H_4_, 4-MeC_6_H_4_, 4-OMeC_6_H_4_, 4-N(Me)_2_C_6_H_4_, 2-chloroquinoline, 2-chloro-6-methylquinoline	R: Et	Bi(NO_3_)_3_·5H_2_O (10 mol%), solvent free, rt/MW	Stirring: 84–94%, 5–10 h, MW: 88–95%, 10–15 min	50
3	**Aldehydes**	R: Et	CeCl_3_·7H_2_O (10 mol%), solvent free, rt	**Aldehydes**	51
	R^1^: H/R^2^: C_6_H_5_, 4-ClC_6_H_4_, 2-ClC_6_H_4_, 4-NO_2_C_6_H_4_, 4-N(Me)_2_C_6_H_4_, 4-OMeC_6_H_4_, 4-OHC_6_H_4_, C_6_H_5_CH=CH, 2-chloroquinoline, 2-chloro-6-methylquinoline			91–95%, 15 min	
	**Ketones**			**Ketones**	
	Cyclopentanone, cyclohexanone			92–95%, 10–12 h	
4	R^1^: H/R^2^: C_6_H_5_, 4-CH_3_C_6_H_4_, 4-OMeC_6_H_4_, 4-NO_2_C_6_H_4_, 3-NO_2_C_6_H_4_, 2-NO_2_C_6_H_4_, 4-ClC_6_H_4_, 2-ClC_6_H_4_, 2,4-di-ClC_6_H_3_, C_6_H_5_CH=CH, 2-furyl, 2-thienyl, Me, Et, *i*-Pr	R: Et	I_2_ (10 mol%), H_2_O, 80 °C	83–97%, 15–120 min	52
5	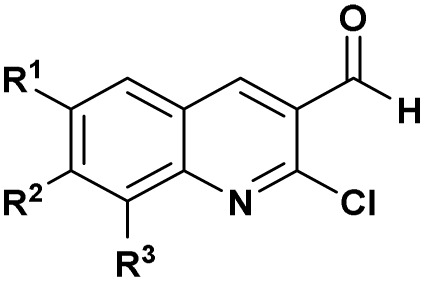	R: Et	TMSCl, rt, solvent free	95–97%, 24–30 min	53
6	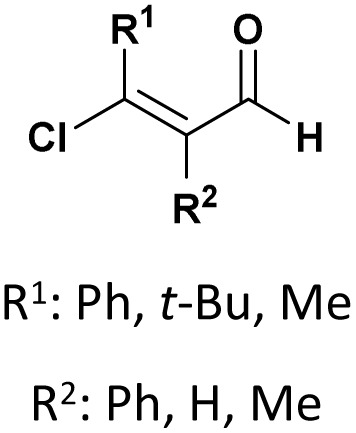	R: Et, Me, Bu	TMSCl (1g), solvent free, rt	75–91%, 24–48 h	54
7	R:^1^ H/R^2^: C_6_H_5_, 4-OHC_6_H_4_, 4-ClC_6_H_4_, C_6_H_5_CH = CH, 4-NO_2_C_6_H_4_, 4-MeC_6_H_4_, 4-OMeC_6_H_4_, Benzo[d][1,3]dioxole, 2-furyl, 2-thienyl, 2-chloroquinoline, Tetrazolo[1,5-*a*]quinoline	R: Et	(1) CSA (10 mol%), Solvent free, rt	(1) 83–92%, 30–60 min	55
			(2) CSA (10 mol%), US, solvent free, rt	(2) 85–93%, 8–20 min	
8	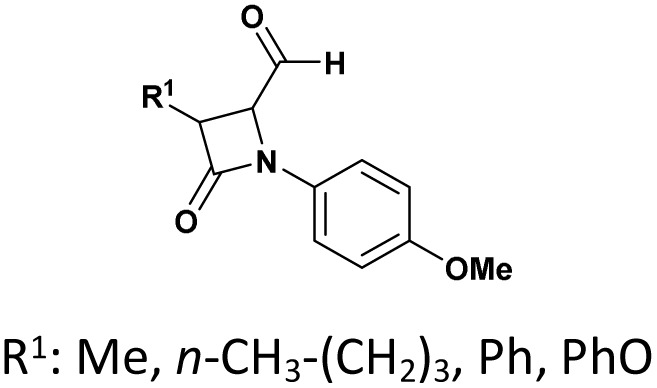	R: Et, Me	Tartaric or fumaric acid (10 mol%), CH_3_CN, reflux, 30 min	Tartaric acid: 41–69%	56
				Fumaric acid: 43–68%	
9	R^1^: H/ R_2_: C_6_H_5_, 4-MeC_6_H_4_, 4-OMeC_6_H_4_, 4-ClC_6_H_4_, 2-ClC_6_H_4_, 3-OHC_6_H_4_, cinnamaldehyde, 4-oxo-4H-chromen-3-carbaldehyde, 6-chloro,7-methyl-4-oxo-4H-chromen-3-carbaldehyde, 2-chloro 3-formyl quinolone, Et, *i*-Pr	R: Et	KH_2_PO_4_ (5 mol%), US, solvent free, rt	48–92%, 5–45 min	57
10	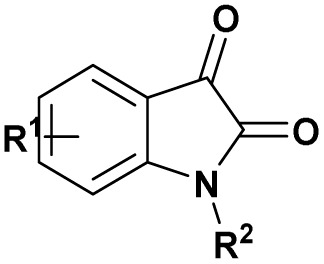	R: Et, Me	β-CD/H_2_O rt	86–94%, 0,5–4 h	58
11	R_1_: H/R_2_: C_6_H_5_, 4-NO_2_C_6_H_4_, 3-NO_2_C_6_H_4_, 4-BrC_6_H_4_, 4-FC_6_H_4_, 4-ClC_6_H_4_, 2-OHC_6_H_4_, 4-MeC_6_H_4_, 4-OMeC_6_H_4_, 2-naphthyl, 2-pyridyl, 2-thienyl, 2-furyl	R: Et	β-CD/H_2_O, 60–70 °C	87–94%, 8–12 h	59
12	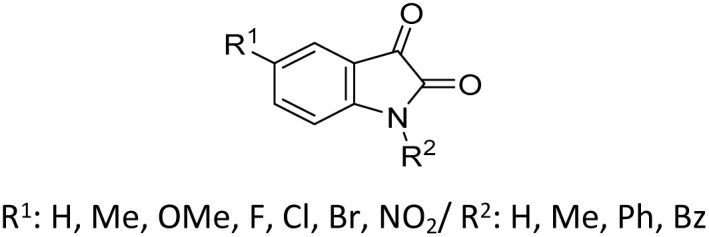	R: Et, Me	PEG-400 (5 mL), solvent free, 50 °C	82–92%, 10 h	60
13	**Aldehydes**	R: Et, Me	US (40 kHz), solvent and catalyst free, rt	84–94%, 10–37 min	61
	R:^1^ H/R^2^: C_6_H_5_, 4-ClC_6_H_4_, 2-ClC_6_H_4_, 4-BrC_6_H_4_, 4-CF_3_C_6_H_4_, 4-OMeC_6_H_4_, 4-N(Me)_2_C_6_H_4_, 4-FC_6_H_4_, 2-NO_2_C_6_H_4_, 4-*i*PrC_6_H_4_, 4-CH_2_C_6_H_4_, 4-CH_2_CH_2_C_6_H_4_, 4-MeC_6_H_4_, isatin				
	**Ketones**				
	R_1_: Ph/R_2_: Ph, Me, Et				

#### 3.1.2. Lewis acid and halogen catalysis

The Abramov reaction has been markedly improved by the use of Lewis acids and halogen-based promoters, which enhance the electrophilicity of carbonyl compounds toward the nucleophilic addition of trialkyl phosphites. Among these systems, ZnBr_2_ (10 mol%)^49^ (entry 1, Table 1) efficiently promotes the reaction under solvent-free conditions, affording α-hydroxyphosphonates in 79–91% yield. The electronic nature of aryl aldehydes plays a decisive role, with moderately electron-withdrawing substituents favoring phosphonate formation over competing pathways.

Building upon this effectiveness, other metal-based Lewis acids have further expanded the scope and efficiency of this transformation. In particular, Bi(NO_3_)_3_·5H_2_O^50^ (10 mol%) catalyzes the direct reaction of aldehydes with triethyl phosphite under solvent-free conditions, delivering the desired products in 84–94% yield after 5–10 h under conventional heating, whereas microwave irradiation reduced the reaction time to 10–15 min and afforded slightly higher yields of 88–95% (entry 2, Table 1).

Likewise, CeCl_3_·7H_2_O^51^ has emerged as a highly effective and versatile catalyst, enabling the rapid conversion of aromatic, heterocyclic, and α, β-unsaturated aldehydes within only 15 min in excellent yields (91–95%) (entry 3, Table 1). Notably, this system also activates less reactive cyclic ketones such as cyclopentanone (92%) and cyclohexanone (95%). In contrast, cyclohexenone undergoes preferential Michael addition rather than the expected 1,2-phosphorylation.

In addition to metallic salts, halogen-mediated activation offers distinct advantages in reaction efficiency and coordination. For instance, it has been demonstrated that 10 mol% of I_2_ (ref. 52) (entry 4, Table 1) in water promotes the coupling of aldehydes with triethyl phosphite at 80 °C to afford α-hydroxyphosphonates in high yields (83–97%). The catalytic role of iodine is attributed to its coordination with the carbonyl oxygen, which enhances the electrophilicity of the aldehyde and facilitates nucleophilic phosphite addition.

Complementarily, chlorinated reagents like TMSCl (entry 5, Table 1) have emerged as powerful halogen-based activators. A solvent-free, room-temperature protocol for the synthesis of α-hydroxyphosphonates from 2-chloroquinoline-3-carbaldehydes was reported,^53^ delivering excellent yields (95–97%) through a highly exothermic process involving a carbonium ion intermediate. Building on this approach, the TMSCl-mediated methodology^54^ was extended to β-chloro-α, β-unsaturated aldehydes, enabling their reaction with trialkyl phosphites under mild, solvent-free conditions. Subsequent acetylation of the intermediate α-hydroxyphosphonates with acetic anhydride and DBU afforded α-acetoxyphosphonates, isolated as mixtures of (*E*)/(*Z*) isomers (entry 6, Table 1).

#### 3.1.3. Organocatalysis and Brønsted acids

Beyond Lewis acids and halogen-based promoters, organocatalysts and Brønsted acids have emerged as efficient and environmentally benign alternatives for the Abramov reaction. These systems typically operate under mild conditions and activate carbonyl compounds through hydrogen bonding or protonation, thereby increasing their susceptibility toward nucleophilic attack by trialkyl phosphites.

Among them, camphorsulfonic acid (CSA, 10 mol%)^55^ has proven to be an effective organocatalyst for the solvent-free synthesis of α-hydroxyphosphonates at room temperature (entry 7, Table 1). The application of ultrasound irradiation markedly enhances the reaction efficiency, shortening reaction times from 30–60 min to only 8–20 min. Mechanistic studies have shown that CSA does not interact with the phosphite reagent, confirming that its catalytic role is limited to carbonyl activation without inducing side reactions.

The scope of Brønsted acid catalysis has also been successfully applied to more structurally complex substrates, including β-lactam-containing α-hydroxyphosphonates. In this context, the use of tartaric acid or fumaric acid (10 mol%)^56^ in refluxing acetonitrile enables efficient condensation within 30 min (entry 8, Table 1). These mild organic acids are particularly advantageous, as they preserve the integrity of the sensitive β-lactam ring, affording the desired products as diastereomeric mixtures readily separable by crystallization.

In line with the development of greener methodologies, potassium dihydrogen phosphate (KH_2_PO_4_)^57^ has been introduced as a mild Brønsted acid catalyst under solvent-free, sonochemical conditions (entry 9, Table 1). This protocol allows for the rapid synthesis of α-hydroxyphosphonates from a broad range of aldehydes at ambient temperature, providing high product purity and improved catalytic efficiency through the combined effects of ultrasound activation and gentle acidity.

#### 3.1.4. Green and alternative activation methods

In recent years, the Abramov reaction has benefited from the development of green and alternative activation strategies that comply with the principles of sustainable chemistry. These approaches aim to reduce environmental impact by avoiding toxic solvents, metal catalysts, and harsh conditions, while preserving high efficiency and broad substrate scope.

An efficient supramolecular catalytic system based on β-cyclodextrin (β-CD) in water was developed by Shankar et *al.*^58^ β-CD promoted the Abramov reaction of substituted isatins with trialkyl phosphites under ambient conditions, delivering α-hydroxyphosphonates in high yields (86–94%) within 0.5–4 h (entry 10, Table 1). The observed catalytic activity was attributed to host–guest inclusion between β-CD and isatin, which facilitates carbonyl activation and enables catalyst recyclability. This approach was later applied to aldehydes, which underwent reaction with triethyl phosphite in aqueous media at 60–70 °C, affording the desired products in 87–94% yield (entry 11, Table 1).^59^ NMR investigations revealed that β-CD enhances the electrophilicity of the carbonyl group through non-covalent hydrogen-bonding interactions.

As an alternative to aqueous systems, polyethylene glycol (PEG-400) has been introduced as a benign, recyclable, and environmentally friendly reaction medium. Nagarapu and co-workers^60^ reported the catalyst-free synthesis of α-oxindole-α-hydroxyphosphonates in PEG-400 at 50 °C, achieving yields of 82–92% within 10 h (entry 12, Table 1). In this case, PEG-400 plays a dual role as both a green solvent and a carbonyl activator.

In addition to solvent-based green systems, physical activation by ultrasound has emerged as a powerful and clean energy source for the Abramov reaction. In this context, our laboratory^61^ developed a sonochemical protocol (40 kHz) enabling the reaction of aryl aldehydes or ketones with trialkyl phosphites under completely solvent and catalyst free conditions at room temperature (entry 13, Table 1). This method affords high-purity α-hydroxyphosphonates within 10–37 min, with products precipitating directly from the reaction medium and being isolated by simple crystallization. The synergy between sonochemistry and solvent-free conditions provides a rapid, energy-efficient, and waste-minimized route to valuable organophosphorus compounds.

#### 3.1.5. Asymmetric synthesis

C-chiral phosphonates represent a biologically significant class of organophosphorus compounds; however, their stereoselective synthesis remains a major challenge in organic chemistry. To date, only a limited number of studies have addressed the preparation of enantioenriched α-hydroxyphosphonates *via* the Abramov reaction.

**Scheme 1:**
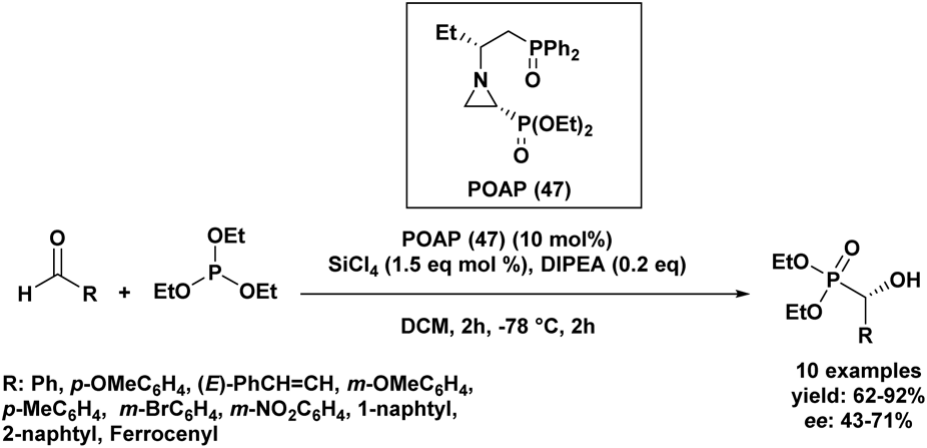
Asymmetric Abramov phosphonylation of aldehydes catalyzed by chiral POAP.

In 2013, a Turkish research group^62^ investigated a series of Lewis bases for their catalytic potential in Abramov-type phosphine additions to aldehydes. They synthesized six novel chiral phosphine oxide aziridinyl phosphonates through a multi-step synthetic route. These chiral Lewis bases were evaluated as catalysts for the enantioselective addition of triethyl phosphite to anisaldehyde in the presence of SiCl_4_. Through reaction optimization, POAP (47) emerged as the most effective catalyst, with diisopropylethylamine (iPr_2_NEt, 0.2 eq.) identified as the optimal additive in dichloromethane (DCM). Adjustments to temperature, the rate of SiCl_4_ addition, and reagent ratios showed minimal influence on enantioselectivity. Among the solvents tested, DCM provided the best balance between yield and selectivity. Further substrate screening demonstrated that the optimized system delivered good to excellent yields (62–92%) across a variety of aldehydes, with moderate, substrate-dependent enantioselectivities ranging from 43% to 71% ee (Scheme 1).

**Scheme 2:**
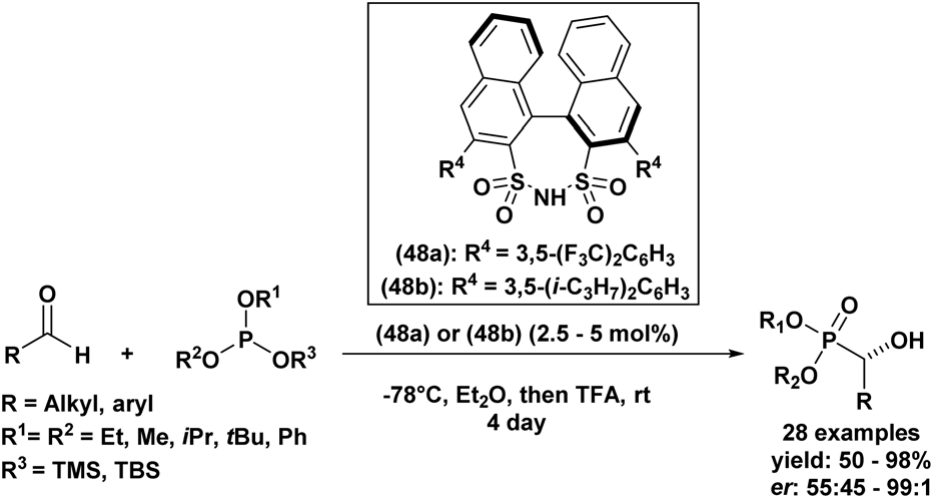
Asymmetric synthesis of α-hydroxyphosphonates catalyzed by chiral disulfonimides.

Another example of an enantioselective Abramov reaction mediated by asymmetric catalysis was described in 2014 by Guin *et al.*^63^ The group reported the use of two chiral disulfonimides (48a) and (48b) as catalysts with a quantity of 2.5 to 5 mol% in order to promote the production of functionalized α-hydroxyphosphonates in a selective manner from several aldehyde derivatives and trisubstituted silyl-based phosphites (Scheme 2). The reaction was privileged by good to excellent yields of the desired compounds (50–98%), in addition to the excellent selectivity with enantiomeric ratios reaching 99 : 1. Further, authors explored the scalability of the reaction by testing it with 1 g of the starting aldehyde, which resulted in the corresponding product without affecting the yield or the enantioselectivity, a fact that confirmed the usability of this method on a gram scale. It is noteworthy that the present procedure is only feasible when using silyl phophites and remained unproductive when employing alkyl phosphites under similar conditions.

**Scheme 3:**
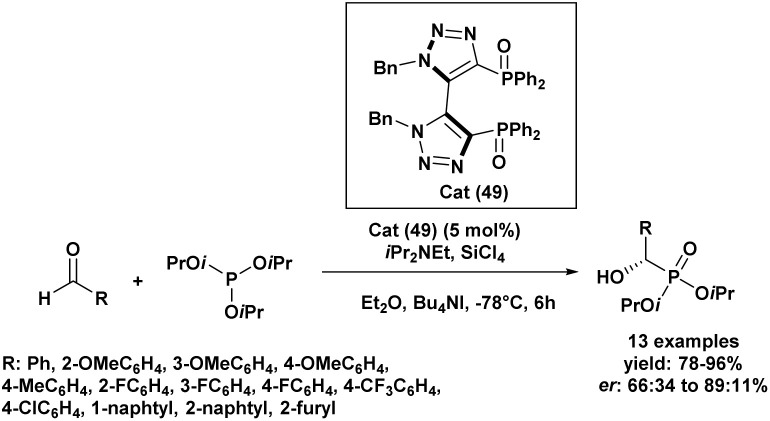
Chiral C_2_-symmetric bis(triazolyl)diphenylphosphine dioxide/SiCl_4_ catalyzed asymmetric hydrophosphonylation of aldehydes.

In 2017, an enantioselective Abramov reaction was achieved using SiCl_4_ in the presence of a chiral C_2_-symmetric diphenylphosphine dioxide organocatalyst incorporating a bis(triazolyl) scaffold (49), enabling the synthesis of α-hydroxyphosphonates (Scheme 3).^64^ Initial experiments in dichloromethane at −78 °C afforded moderate yields and enantioselectivities, but the addition of tetrabutylammonium iodide significantly improved both parameters. Subsequent solvent screening identified diethyl ether as the most effective medium, enabling a 91% yield with an enantiomeric ratio (er) of 84.5 : 15.5. Among various trialkyl phosphites, triisopropyl phosphite gave the best performance, yielding 94% of the desired product with an (er) of 88.5 : 11.5. Catalyst loading had a limited effect, although lower amounts required extended reaction times. Of all additives tested, tetrabutylammonium iodide proved to be the most efficient. The optimized protocol was then applied to a diverse range of aryl aldehydes, yielding the corresponding α-hydroxyphosphonates in 78–96% with high enantioselectivities (up to 89 : 11), regardless of the electronic nature or substitution pattern on the aromatic ring. Benzaldehyde derivatives with electron-donating groups provided excellent outcomes (er 85.5 : 14.5 to 88.5 : 11.5), and similar performance was observed for electron-withdrawing substituents such as –F and –CF_3_. In contrast, *p*-chlorobenzaldehyde showed reduced selectivity despite a high yield (91%). Sterically hindered *ortho*-substituted aldehydes led to diminished yields (78–81%) and lower enantioselectivities (67.5 : 32.5 to 72 : 28), underscoring the steric sensitivity of the catalytic system. Notably, 2-naphthaldehyde afforded excellent results (95% yield, 88.5 : 11.5 er), while 2-furaldehyde proved incompatible under the reaction conditions.

### 3.2. *Via* Pudovik reaction conditions

#### 3.2.1. General mechanism and reaction features

The Pudovik reaction, named after the Russian chemist Alexander N. Pudovik in 1950,^26^ involves the addition of dialkyl phosphites to aldehydes or ketones to produce α-hydroxyphosphonates. This transformation is a valuable tool in organic synthesis, as it enables the preparation of compounds containing both phosphorus and oxygen functional groups.

The mechanism of the Pudovik reaction^48^ is proposed to begin with the deprotonation of dialkyl phosphite (A′) to generate dialkyl hydrogen phosphite (A), a highly nucleophilic species. This nucleophile subsequently attacks the carbonyl carbon of the aldehyde or ketone (B), forming a tetrahedral intermediate (C).

**Fig. 10:**
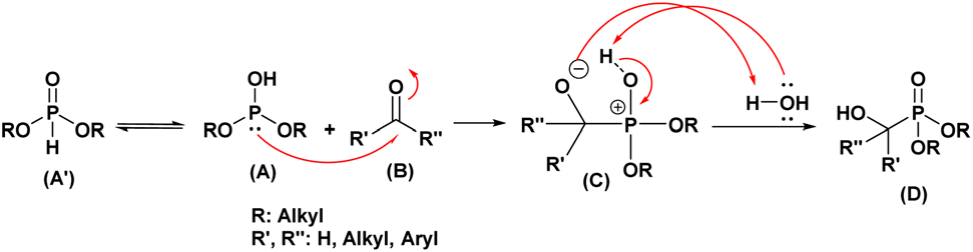
Mechanistic proposal for the Pudovik reaction.

In aqueous solution, this intermediate undergoes proton transfer, leading to the formation of the desired α-hydroxyphosphonate product (D), as illustrated in Fig. 10. Overall, the reaction follows a nucleophilic addition pathway.

Over the years, several variations and modifications of the Pudovik reaction have been developed to enhance its scope and selectivity. Some examples are presented in the following table.

#### 3.2.2. Base-catalyzed systems

Base-catalyzed systems represent one of the most extensively explored and reliable strategies for promoting the Pudovik reaction. Their efficiency originates from the activation of the P–H bond of dialkyl phosphites, which undergoes deprotonation in the presence of a base to generate a highly nucleophilic phosphonate anion. This reactive species readily adds to electrophilic carbonyl compounds, leading to the formation of α-hydroxyphosphonates after protonation. Owing to this straightforward activation mode, base catalysis is compatible with a wide range of substrates and reaction environments, including solvent-free conditions, mild temperatures, and heterogeneous catalytic systems.

Within this category, inorganic bases have attracted considerable interest owing to their robustness and ease of integration into diverse synthetic protocols. In this context, calcium phosphate-based materials, such as sodium-modified hydroxyapatite (Na-HAP),^65^ have proven particularly effective for solvent-free Pudovik reactions at room temperature (entry 1, Table 2). In their study, aldehydes reacted rapidly at ambient temperature in the presence of 1 g of Na-HAP, affording excellent yields (78–98%) within 1–5 min, whereas ketones required longer reaction times (60–90 min) to achieve comparable yields. Notably, the Na-HAP catalyst could be readily recovered, washed, and reused several times without any significant loss of activity, underscoring its practical value for sustainable synthesis.

**Table 2:** Some examples of Pudovik reaction for α-hydroxyphosphonates

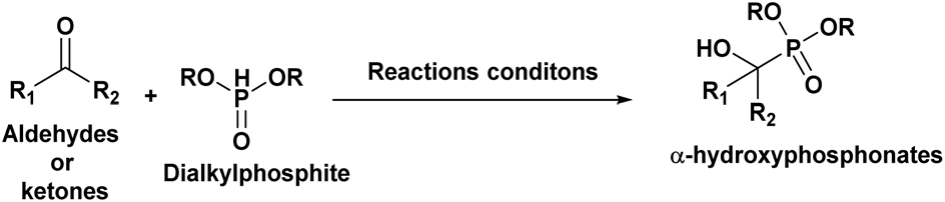					
Entry	Aldehydes or ketones	Dialkylphosphite	Reaction conditions	Yield %/Time (min)	Ref.
1	**Aldehydes**	R: Me, Et	1g Na-HAP, solvent free, rt	**Aldehydes**	65
	R^1^: H/R^2^: Ph, 4-ClC_6_H_4_, CH = CHC_6_H_6_, 3-NO_2_C_6_H_4_, 4-OMeC_6_H_4_, 3-OMeC_6_H_4_			78–98%, 1–5 min	
	**Ketones**			**Ketones**	
	R^1^: H/ R^2^: Ph, 4-OMeC_6_H_4_, 4-NO_2_C_6_H_4_			78–98%, 60–90 min	
2	**Aldehydes**	R: Me, Et	(Na@FAP) (1g), solvent free, rt	**Aldehydes**	66
	R:^1^ H/R^2^: Ph, 4-ClC_6_H_4_, CH=CHC_6_H_5_, 3-NO_2_C_6_H_4_, 4-OMeC_6_H_4_, 3-OMeC_6_H_4_			80–98%, 1 min	
	**Ketones**			**Ketones**	
	R^1^: H/ R^2^: Ph, 4-OMeC_6_H_4_, 4-NO_2_C_6_H_4_			75–98%, 60–90 min	
3	R^1^: H/R^2^: C_6_H_5_, 4-OMeC_6_H_4_, 4-MeC_6_H_4_, 4-NO_2_C_6_H_4_, 4-ClC_6_H_4_	R: Me, Et, Ph	MW, Na_2_CO_3_ (0.75 eq.), solvent free, 110 °C	71–88%, 20 min	67
4	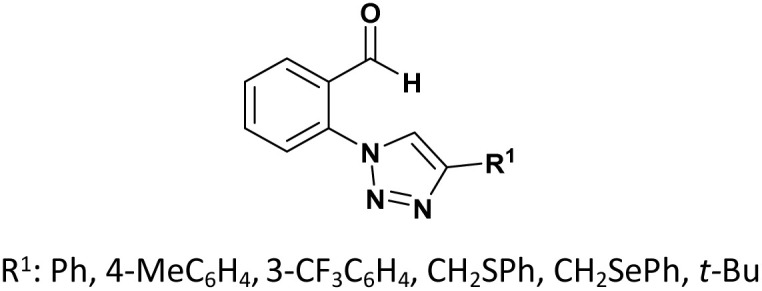	R: Me, Et, Bu	Na_2_CO_3_ (10 mol%), solvent-free, rt	76–93%, 3 h	68
5	R:^1^: H/R^2^: C_6_H_5_, 4-NO_2_C_6_H_4_, 3-NO_2_C_6_H_4_, 2-NO_2_C_6_H_4_, 4-MeC_6_H_4_, 3-MeC_6_H_4_, 2-MeC_6_H_4_, 4-BrC_6_H_4_, 3-BrC_6_H_4_, 4-ClC_6_H_4_, 2-ClC_6_H_4_, 2,6-di-ClC_6_H_3_, 4-CNC_6_H_4_, 3-CNC_6_H_4_, 4-EtC_6_H_4_, 4-FC_6_H_4_, 3-FC_6_H_4_, 4-OMeC_6_H_4_, 2-furyl, 2-thienyl, 2-pyridyl, 3-pyridyl, 4-pyridyl, 2,3,4-tri-OMe-C_6_H_4_, 1-naphthyl, 2-naphthyl, 9-anthryl, cinnamyl, isobutyl	R: Et, *n*-Bu	Ba(OH)_2_·8H_2_O (2 mol%), THF, rt	72–99%, 15 min	69
6	R^1^: H/R^2^: C_6_H_5_, 4-ClC_6_H_4_, 4-MeC_6_H_4_, 4-OMeC_6_H_4_, 4-*i*PrC_6_H_4_, 3-NO_2_C_6_H_4_, 4-CNC_6_H_4_, CH=CH–C_6_H_5_, benzo[d][1,3]dioxole, 3,4-di-OMeC_6_H_3_, 4-CH_2_=CH–CH_2_O–C_6_H_4_, 4-OBz-C_6_H_4_, 2-furyl, 2-thienyl, isopropyl, cyclohexane	R: Me, Et, i-Pr	K_3_PO_4_ (5 g mol^−1^%), solvent free, rt	20–98%, 4 min-24 h	70
7	R^1^: Ph, 4-MeC_6_H_4_, 4-OMeC_6_H_4_/R^2^: CH_2_CH_2_CN, 3-methylbutanenitrile	R: Me, Et	MgO (2 eq.), solvent-free, 25 °C	70–82%, 1-6 h	71
8	R^1^: H/ R^2^: Ph, 4-ClC_6_H_4_, 4-NO_2_C_6_H_4_, 4-MeC_6_H_4_, 2,4,6-tri-MeC_6_H_2_, CH_2_-*i*Pr, Et, *i*Pr, C(CH_2_CH_3_)_2_, 3-pyridyl, 3-quinoline, 2-thienyl, CH=CH–C_6_H_4_	R: Et	Eco-MgZnOx, solvent-free, 50–70 °C	75–99%, 3 h	72
9	R^1^: H/ R^2^: Ph, 4-MeC_6_H_4_, 4-OMeC_6_H_4_, 3-OHC_6_H_4_, 4-OH-3-OMe-C_6_H_3_, 4-ClC_6_H_4_, 2-ClC_6_H_4_, 2,6-di-ClC_6_H_3_, 2-NO_2_C_6_H_4_, 3-NO_2_C_6_H_4_, CH = CH–C_6_H_4_, 4-(Me)_2_NC_6_H_4_, 4-(Et)_2_NC_6_H_4,_ C_6_F_5_, Quinolin-2-yl	R: Et	Nano-TiO_2_ (10 mol%), MW, solvent-free	80–95%, 4–10 min	73
10	R^1^: H/R^2^: 2-Bromothienyl, 2-chloro-6-fluoro-phenyl, pyridin-4-yl, 2-nitrothienyl, 4-hydroxy-3-nitrophenyl, 4-chloro-3-nitrophenyl, 2-bromo-4-fluorophenyl, 4-trifluoromethoxyphenyl, 3,5-dichloro-2-hydroxyphenyl, 3-bromo-5-chloro-2-hydroxyphenyl, 2-chloro-5-hydroxyphenyl, 4-(diethylamino)-2-hydroxyphenyl	R: Et	Nano-ZnO sheets (20 mol%), sonication: EtOH, 50 °C or conventional method: EtOH, 50 °C	Ultrasonic method: 72–91%, 20–45 min conventional method: 66–88%, 4–8 h	74
11	R^1^: Me/R^2^: CH_2_PO(OEt)_2_	R: Me, Et, *n*-Bu, i-Pr, Bn	Al_2_O_3_/KF, 25 °C	62–80%, 72 h	75
12	R^1^: H, CH_3_	R: Me, Et, Bu	TEA (0.5 eq.), EtOAc, 0 °C, 12 h or Al_2_O_3_/KF, 25 °C, 24h or MW, Na_2_CO_3_, solvent-free, 110 °C, 2 h	65–93%	76
	R^1^: CH_3_				
13	R^1^: H/ R^2^: C_6_H_5_, 4-ClC_6_H_4_, 2-ClC_6_H_4_, 4-NO_2_C_6_H_4_, 3-NO_2_C_6_H_4_, 2-NO_2_C_6_H_4_, 4-OMeC_6_H_4_, 4-MeC_6_H_4_, 4-*i*PrC_6_H_4_, 3,4-di-OMeC_6_H_3_, 4-N(Me)_2_C_6_H_4_, anthracene, 2-phenylpyridine, (benzyloxy)benzene	R: Et	Piperazine, grinding, solvent free, rt	78–96%, 2–10 min	77
14	R^1^: H/R^2^: Ph, 2-NO_2_C_6_H_4_, 4-OMeC_6_H_4_, 4-MeC_6_H_4_, 4-BrC_6_H_4_, 2-ClC_6_H_4_, 3-BrC_6_H_4_, 4-ClC_6_H_4_, 4-FC_6_H_4_, 3,4-di-ClC_6_H_3_, 2,3-di-ClC_6_H_3_, 2-OHC_6_H_4_, 4-CHOC_6_H_4_, 1-naphthyl, CH=CHC_6_H_5_, 2-furyl	R: Me	MNPs-guanidine (15 mg), solvent-free, 80 °C	62–98%,75–240 min	78
15	R^1^: H/R^2^: Ph, 4-OMeC_6_H_4_, 3-OMeC_6_H_4_, 2-OMeC_6_H_4_, 2-ClC_6_H_4_, 2-furyl, 2-thienyl, *n*-pentyl, i-butyl, cyclohexyl, (*E*)-PhCH=CH, 2-pyridyl, 4-CNC_6_H_4_	R: Et	PS-BEMP (5 mol%), solvent-free, 30 °C	94–95%, 2,5–36 h	79
16	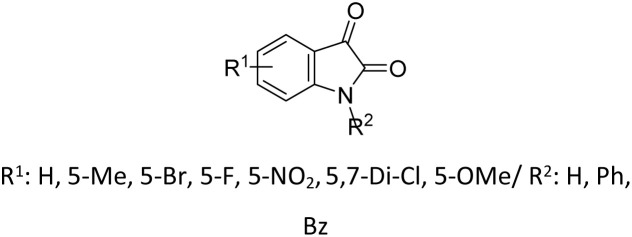	R: Me, Et	MNP-TBD (40 mg), solvent-free, 60 °C	79–93%, 5–8 h	80
17	**Aldehydes**	R: Me, Et	TEA, (CH_3_)_2_CO, reflux	**Aldehydes**	81
	R:^1^ H/R^2^: Ph, 4-ClC_6_H_4_, 4-NO_2_C_6_H_4_, 2-NO_2_C_6_H_4_, 4-MeC_6_H_4_, 3,4-di-OMeC_6_H_4_			78–95%, 5–120 min	
	**Ketones**			**Ketones**	
	R^1^: Me/R^2^: Ph, 4-NO_2_C_6_H_4_			40–81%, 45–180 min	
18	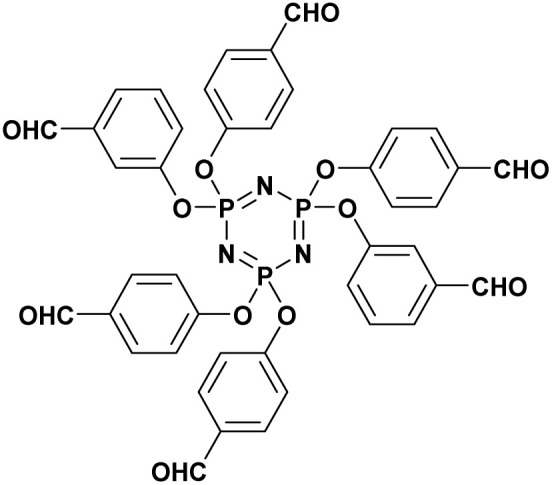	R: Et	TEA, solvent-free, rt	95.4%, 70 min	82
19	R^1^: H/R^2^: 2-ClC_6_H_4_, 2-BrC_6_H_4_, 3-BrC_6_H_4_, 4-BrC_6_H_4_, 2-Cl-6-F-C_6_H_3_, 2-F-4-Br-C_6_H_3_, 2-MeC_6_H_4_, 3,5-di-MeC_6_H_3_, 4-MeC_6_H_4_, 2-OMeC_6_H_4_, 4-OMeC_6_H_4_, 3,5-di-OMeC_6_H_3_, 3,4,5-tri-OMeC_6_H_2_, 4-OEtC_6_H_4_, 4-*i*PrC_6_H_4_, 4-NO_2_C_6_H_4_, 2-NO_2_C_6_H_4_, AnC_6_H_4_, PyC_6_H_4_, FfC_6_H_4_, PPyC_6_H_4_	R: Et	ChOH (2.5 mL), solvent-free, rt	90–98%, 4–10 min	83
20	R_1_: H/R_2_: Ph, 1-naphthyl, 2-FC_6_H_4_, 2-BrC_6_H_4_, 4-ClC_6_H_4_, 4-OMeC_6_H_4_, 4-NO_2_C_6_H_4_, 4-CNC_6_H_4_, 4-(Me)_2_NC_6_H_4_, 3,5-di-OMeC_6_H_3_, 4-OHC_6_H_4_, 2-furyl, 2-thienyl	R: Et	H_6_P_2_W_18_O_62_·14H_2_O (1 mol%), solvent-free, rt	88–97%, 10 min	84
21	R^1^: H/R^2^: Ph, 4-BrC_6_H_4_, 4-NO_2_C_6_H_4_, 3-NO_2_C_6_H_4_, 2-NO_2_C_6_H_4_, 4-ClC_6_H_4_, 2-ClC_6_H_4_, 4-MeC_6_H_4_, 4-OHC_6_H_4_, 4-OMeC_6_H_4_	R: Et	MW (100–120 W), solvent-free, 90–100 °C	79–95%, 10–20 min	85

Along the same lines, sodium-modified fluorapatite (Na@FAP) was subsequently developed as an efficient inorganic base for the synthesis of α-hydroxyphosphonates (entry 2, Table 2).^66^ Under solvent-free conditions at ambient temperature, this calcium phosphate–based catalyst effectively promoted the Pudovik reaction between dialkyl phosphites and carbonyl compounds, affording the desired products in high yields (78–95%) within 30–90 min. The methodology also benefits from straightforward product isolation and good catalyst recyclability, further reinforcing the potential of apatite-derived inorganic bases for sustainable organophosphorus synthesis.

Alkali metal carbonates, with sodium carbonate (Na_2_CO_3_) being the most prominent example, form another important class of inorganic bases. Under solvent-free microwave irradiation, Na_2_CO_3_ (0.75 equiv., 110 °C) has been shown to efficiently promote the condensation of aromatic aldehydes with dialkyl phosphites and diphenylphosphine oxide (entry 3, Table 2),^67^ leading to the formation of α-hydroxybenzylphosphonates and α-hydroxybenzylphosphine oxides in good yields (71–88%) within 20 min. In subsequent investigations, Na_2_CO_3_ used at a lower loading (10 mol%) under comparable solvent-free Pudovik conditions afforded moderate to excellent yields (76–93%) over a wide substrate scope, encompassing esters, ketones, and amides (entry 4, Table 2).^68^

Stronger inorganic bases, such as barium hydroxide octahydrate [Ba(OH)_2_·8H_2_O], have also been successfully employed in homogeneous media (entry 5, Table 2).^69^ In this case, the reaction proceeds rapidly at room temperature in THF, delivering α-hydroxyphosphonates in quantitative yields within 15 minutes. The high basicity and solubility of barium hydroxide facilitate efficient P–H bond activation, although its homogeneous nature contrasts with the recyclability advantages offered by heterogeneous systems.

Moderately strong bases, such as potassium phosphate (K_3_PO_4_), have been successfully employed under solvent-free conditions (entry 6, Table 2).^70^ Aromatic aldehydes react efficiently within 4–8 minutes, providing excellent yields (92–98%), whereas aliphatic aldehydes display reduced reactivity, requiring significantly longer times (up to 24 h) and affording lower yields (20–30%), reflecting substrate-dependent electrophilicity effects. The catalytic efficiency of K_3_PO_4_ is attributed to its balanced basicity, which is sufficient to activate phosphites while remaining compatible with sensitive functional groups. This methodology also benefits from the commercial availability of the catalyst, simple operational procedures, and the elimination of conventional work-up steps.

Metal oxides constitute another important subclass of inorganic bases in Pudovik chemistry. Magnesium oxide (MgO), used under solvent-free conditions at 25 °C (entry 7, Table 2),^71^ effectively catalyzes reactions involving aldehydes and functionalized ketones, including γ-ketonitriles. The basic O^−2^ sites on the MgO surface play a key role in phosphite deprotonation, while the solid support enables easy catalyst handling and reuse. The resulting α-hydroxyphosphonates have also been evaluated for biological activity, illustrating the synthetic relevance of this methodology beyond purely methodological studies.

Grison's team, in a 2022 Molecules publication,^72^ introduced Eco-MgZnOx, a novel class of environmentally friendly catalysts. These catalysts are generated by a controlled thermal treatment of zinc-rich biomass from plants like *Arabidopsis halleri*, eliminating the need for chemical activation and resulting in green, basic materials with a unique polymetallic structure. These ecocatalysts demonstrated outstanding performance in the hydroxyphosphonylation reaction, efficiently producing various hydroxyphosphonates (entry 8, Table 2) in high yields under mild, solvent-free conditions with short reaction times (3–5 hours) at modest heating (50–70 °C). The synergistic effect of the MgZnO components was theoretically confirmed, and the catalyst demonstrated excellent reusability over multiple cycles.

#### 3.2.3. Lewis acid and halogen catalysis

Lewis acid catalysis has proven to be an efficient strategy for promoting the Pudovik reaction by activating carbonyl compounds toward nucleophilic addition of phosphite reagents. In this context, nano-sized metal oxides have attracted considerable attention due to their enhanced surface area and pronounced Lewis acidic character. Nano-TiO_2_ has been successfully applied as a heterogeneous Lewis acid catalyst under solvent-free, microwave-assisted conditions for the synthesis of α-hydroxyphosphonates from a wide range of aromatic and heteroaromatic aldehydes (entry 9, Table 2).^73^ The reactions proceed rapidly, affording the desired products within 4–10 minutes. Substituent effects revealed increased reactivity in the presence of electron-withdrawing groups, whereas electron-donating substituents and ortho steric effects resulted in reduced yields, consistent with a carbonyl activation mechanism mediated by surface Lewis acidic sites.

Similarly, ZnO nanosheets have been employed as Lewis acid catalysts in the Pudovik reaction under ultrasound irradiation or conventional heating (entry 10, Table 2).^74^ These catalysts efficiently promote the addition of diethyl phosphite to aromatic and heterocyclic aldehydes, leading to the formation of α-hydroxyphosphonates with high efficiency. The Lewis acidic Zn^+2^ centers are believed to play a key role in carbonyl activation, while the nanostructured morphology enhances catalytic performance.

Beyond discrete metal oxide catalysts, surface-mediated systems combining Lewis acid activation and heterogeneous catalysis have also been reported. The Al_2_O_3_/KF system enables the addition of dialkyl phosphites or diphenylphosphine oxide to activated carbonyl substrates under mild conditions (entry 11, Table 2), allowing access to structurally diverse α-hydroxyphosphonate derivatives.^75^ Structural and conformational features of the resulting products were elucidated using spectroscopic and crystallographic techniques.

In a related but distinct study, the Pudovik reaction of acetaldehyde and acetone with phosphite reagents was explored using multiple activation modes, including Lewis acid surface catalysis with Al_2_O_3_/KF, alongside solution-phase and microwave-assisted methodologies (entry 12, Table 2).^76^ These investigations further highlight the versatility of Lewis acid–mediated and surface-assisted approaches in expanding the scope of α-hydroxyalkylphosphonates and phosphine oxide derivatives, while providing valuable insight into their solid-state organization and downstream reactivity.

#### 3.2.4. Organocatalysis and Brønsted acid catalysis

Organocatalysis and Brønsted acid catalysis have emerged as efficient and versatile alternatives to metal-based systems in the Pudovik reaction. These approaches rely either on organic bases capable of activating the P–H bond of dialkyl phosphites through deprotonation, or on Brønsted acids that enhance the electrophilicity of carbonyl compounds *via* protonation or hydrogen-bonding interactions. The absence of metal species, combined with operational simplicity and compatibility with solvent-free conditions, makes these strategies particularly attractive from both synthetic and environmental perspectives.

Early examples of organocatalytic Pudovik reactions demonstrated that simple nitrogen-containing bases could efficiently promote hydrophosphonylation under mild conditions. In a grinding-assisted, solvent-free protocol, aldehydes were shown to react rapidly with diethyl phosphite at room temperature in the presence of piperazine as a catalyst, delivering α-hydroxyphosphonates in high yields [78–98%] in 2 to 10 minutes (entry 13, Table 2).^77^ The catalytic efficiency of piperazine is associated with its bifunctional basic sites, which facilitate P–H bond deprotonation while maintaining effective interaction with the carbonyl substrate.

The development of heterogeneous organocatalysts represented a significant advance toward efficient catalyst recovery and reuse. In this context, an Iranian research grou^78^ reported the synthesis and characterization of a magnetically heterogeneous nanocatalyst based on guanidine immobilized on Fe_3_O_4_ nanoparticles (MNPs–Guanidine), designed for the solvent-free Pudovik reaction (entry 14, Table 2). This system, recognized as the first magnetically recoverable guanidine-based catalyst, efficiently promoted the reaction between aldehydes and dimethyl phosphite at 80 °C, affording α-hydroxyphosphonates and α-acetoxyphosphonates with good to high conversions. The catalyst could be readily separated using an external magnetic field and reused over several cycles with only a minor decrease in activity, highlighting its potential for practical and industrial applications.

Strong organic superbases have further expanded the scope of organocatalysis in this transformation. A polymer-supported BEMP (PS-BEMP)^79^ catalyst was successfully applied to the hydrophosphonylation of both aromatic and aliphatic aldehydes with diethyl phosphite under solvent-free conditions at moderate temperature (entry 15, Table 2). The reaction, performed using equimolar amounts of the starting materials at 30 °C, afforded the corresponding α-hydroxyphosphonates within reaction times ranging from 2.5 to 36 h, and the products could be isolated with minimal solvent consumption. The PS-BEMP catalyst efficiently promoted the Pudovik reaction, providing high yields while maintaining a low environmental impact, as reflected by favorable E-factor values. Furthermore, the methodology was successfully translated to a continuous-flow reactor, demonstrating its scalability and leading to a significant improvement in sustainability, with E-factor reductions of 92.7–96.3%.

In 2014, a novel magnetic nanocatalyst, γ-Fe_2_O_3_-immobilized 1,5,7-triazabicyclo[4.4.0]dec-5-ene (MNP-TBD),^80^ was utilized to develop an efficient synthesis of α′-oxindole-α-hydroxyphosphonates (entry 16, Table 2). This nanoparticle-based catalyst facilitated the reaction between isatins and dialkyl phosphate under solvent-free conditions at 60 °C. The catalyst demonstrated excellent recoverability *via* an external magnet and was reused up to six times without significant activity decline.

In addition to strong amidine- and guanidine-type superbases, tertiary amines have been extensively investigated as mild and versatile organocatalysts for the Pudovik reaction. In this context, Keglevich *et al.*^81^ reported an environmentally benign protocol employing triethylamine (TEA) as the catalyst for the hydrophosphonylation of various substituted benzaldehydes and ketones with dialkyl phosphites (entry 17, Table 2). The reactions were conducted in refluxing acetone and proceeded efficiently, affording the corresponding α-hydroxyphosphonates in good to excellent yields (40–96%) within short reaction times (5–180 min). Product isolation was straightforward and achieved by simple crystallization from pentane, further enhancing the operational simplicity of the method.

Beyond simple carbonyl substrates, the applicability of TEA catalysis was successfully extended to structurally complex systems. A two-step synthetic strategy was established for the preparation of hexa-(4-diethylphosphatehydroxylmethyl phenoxy) cyclotriphosphazene (HDHPCP). The key transformation relied on a solvent-free Pudovik reaction of the corresponding hexa-aldehyde intermediate, hexa-(4-aldehydephenoxy) cyclotriphosphazene (HAPCP) (entry 18, Table 2).^82^ A systematic comparison of catalysts demonstrated that triethylamine outperformed inorganic bases such as KF and K_2_CO_3_, affording superior reaction rates and higher yields. Under optimized conditions, using a 1 : 5 molar ratio of HAPCP to TEA, the target compound was obtained in an excellent yield of 95.4% within 70 min.

Choline hydroxide has been established as a highly effective basic ionic liquid catalyst for the synthesis of α-hydroxyphosphonates (entry 19, Table 2).^83^ Under solvent-free conditions at room temperature, choline hydroxide efficiently promotes the hydrophosphonylation of a broad range of aldehydes, including aromatic, fused aromatic, and heterocyclic substrates, *via* a straightforward nucleophilic addition of diethyl phosphite to the carbonyl group. This environmentally benign protocol provides a direct and efficient route to α-hydroxyphosphonates, affording excellent yields (90–98%) without the need for external solvents or harsh reaction conditions. The high catalytic efficiency is attributed to the ionic Brønsted basic character of choline hydroxide, which enables effective activation of both reaction partners through strong electrostatic interactions.

Complementary to base-catalyzed systems, Brønsted acid activation has emerged as an efficient alternative for the Pudovik reaction. Our research group reported in 2020 a rapid and high-yielding protocol for the synthesis of structurally diverse α-hydroxyphosphonates *via* a Brønsted acid–promoted Pudovik reaction (entry 18, Table 2).^84^ This methodology employs heteropoly acids (HPAs, 1 mol%) as highly effective catalysts for the solvent-free coupling of diethyl phosphite with various aldehydes. The reactions proceeded smoothly at room temperature, reaching completion within 10 min and affording the desired products in excellent yields (90–97%). The remarkable catalytic efficiency of HPAs is attributed to their strong Brønsted acidity and well-defined polyoxometalate structures, which enable efficient carbonyl activation and rapid C–P bond formation.

#### 3.2.5. Green and alternative activation methods

Beyond catalyst-driven strategies, alternative activation techniques have emerged as powerful and sustainable tools for the Pudovik reaction. In this context, a microwave-assisted, solvent- and catalyst-free protocol was reported, enabling the efficient synthesis of α-hydroxyphosphonates directly from aldehydes and diethyl phosphite (entry 9, Table 2).^85^ Under optimized microwave irradiation conditions (100–120 W) at 90–100 °C, the reaction proceeded rapidly, reaching completion within 10–20 min and affording the desired products in high yields (79–95%) with excellent purity. The enhanced efficiency is attributed to rapid and homogeneous dielectric heating, which accelerates C–P bond formation while avoiding excessive energy consumption. This approach eliminates the need for solvents and catalysts, simplifies work-up, and significantly reduces reaction times, underscoring the potential of microwave activation as a clean and practical alternative for sustainable hydroxyphosphonate synthesis.

#### 3.2.6. Asymmetric synthesis

Asymmetric Pudovik catalysis enables the transformation of prochiral carbonyl compounds into optically active organophosphorus scaffolds. Despite the inherent difficulty of inducing asymmetry in this reaction, modern chiral catalytic systems have made it possible to construct stereogenic centers with high precision, which are essential for the synthesis of complex natural products and therapeutic agents.^86^

**Scheme 4:**
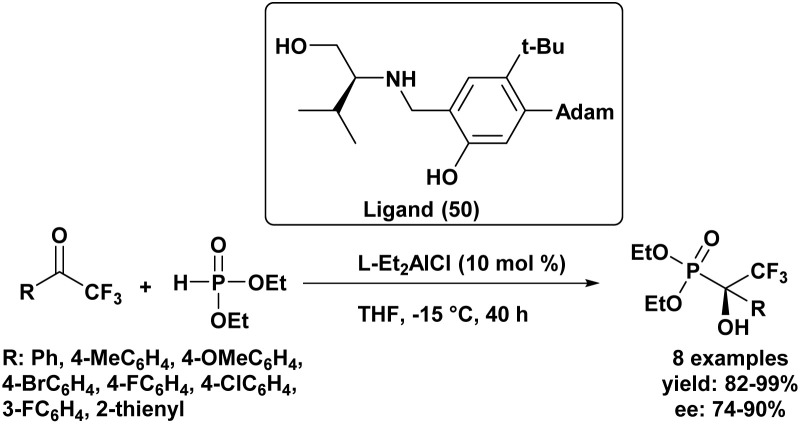
Chiral aluminum(iii)-catalyzed asymmetric hydrophosphonylation of trifluoromethyl ketones.

The first highly enantioselective hydrophosphonylation of trifluoromethyl ketones was reported in 2010 (Scheme 4).^87^ Using a chiral hydrogenated tridentate Schiff base–aluminum(iii) complex as catalyst (50), the reaction proceeded efficiently in THF at −15 °C over 40 h, affording quaternary α-hydroxy trifluoromethyl phosphonates in excellent yields (82–99%) and high enantioselectivities (72–90% ee). Notably, this methodology effectively suppressed the phospha-Brook rearrangement and related side reactions, highlighting its potential for the large-scale synthesis of chiral quaternary α-hydroxyphosphonates.

**Scheme 5:**
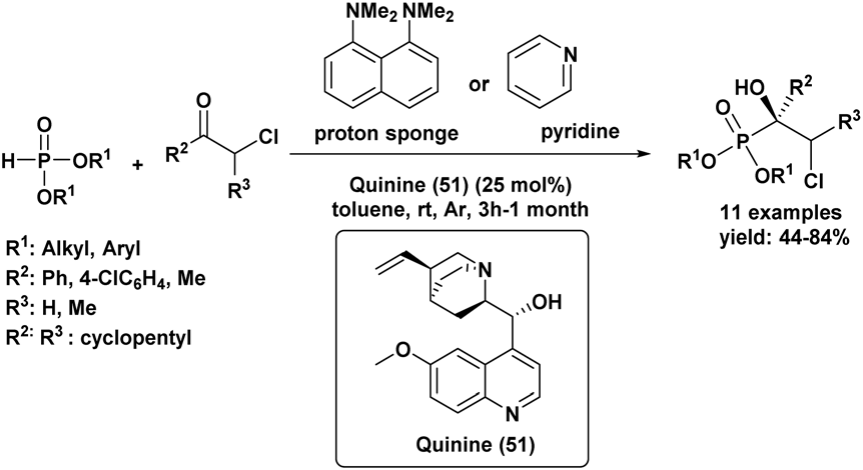
Quinine/base-promoted regioselective modified Pudovik reaction for tertiary α-hydroxyphosphonates.

A Portuguese research group^88^ developed an efficient organocatalyzed method for the synthesis of tertiary α-hydroxyphosphonates *via* a highly regio and stereoselective modified Pudovik reaction (Scheme 5). This transformation involves the addition of dialkyl/diaryl phosphites to α-haloketones under mild conditions, leading to the formation of β-chloro-α-hydroxyphosphonates in good yields. The reaction tolerates a wide variety of substrates, including aliphatic, aromatic, and cyclic ketones, showcasing a broad substrate scope. Enantioselective variants of the reaction, employing quinine (51) as a chiral catalyst and a stoichiometric amount of racemic proton sponge as the base under specific conditions (phosphite/ketone/quinine/proton sponge or pyridine 1 : 1.5 : 0.1 : 1 in toluene, room temperature), produced desired compounds with moderate enantiomeric excesses (up to 40%). The β-chloro substituent introduced during the reaction provides a versatile handle for further functionalization, enhancing the synthetic utility of these α-hydroxyphosphonates, which are of significant interest due to their potential biological activity.

**Scheme 6:**
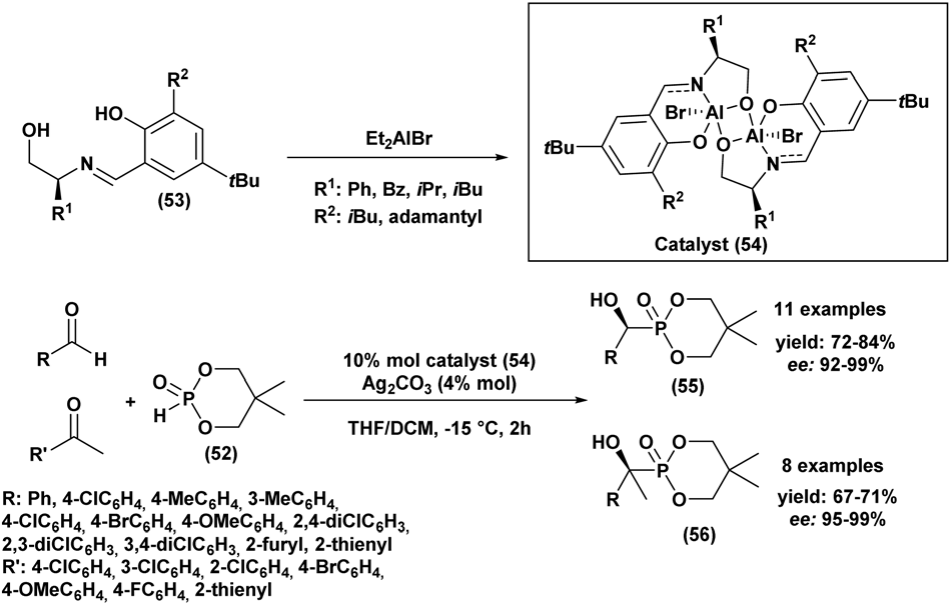
Asymmetric hydrophosphonylation of aldehydes and ketones catalyzed by Al(iii) complex.

In 2012, Wang *et al.*^89^ reported an easy and practical method for the asymmetric synthesis of chiral cyclic α-hydroxyphosphonates (55) and quaternary cyclic *α*-hydroxyphosphonates (56) *via* the enantioselective hydrophosphonylation of ketones and aldehydes, using Schiff base aluminum(iii) complexes as catalysts (54) (Scheme 6). The reaction rate was significantly enhanced by the addition of silver carbonate, allowing the transformation to proceed efficiently and reach completion within 2 hours. The resulting aldehyde and methyl ketone adducts were obtained in good yields (67–84%) and excellent enantioselectivities (92–99% *ee*). Tridentate Schiff base ligands (53) reacted *in situ* with Et_2_AlBr to form aluminum complexes that effectively catalyzed the asymmetric hydrophosphonylation of aldehydes and ketones (Scheme 6). The enantioselectivity was strongly influenced by the nature of the R_1_ and R_2_ substituents on the ligand. Ligands bearing bulky groups, such as R_1_ = *tert-*butyl and R_2_ = adamantyl, provided the highest enantioselectivities.

**Scheme 7:**
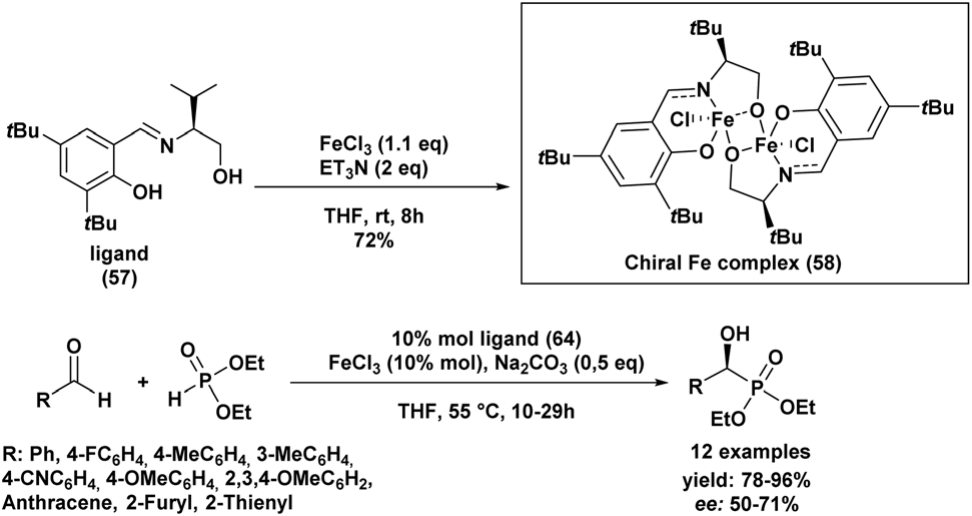
Asymmetric hydrophosphorylation of aldehydes catalyzed by a chiral iron complex.

In the same year, Sekar and Muthupandi^90^ reported the synthesis and application of a chiral iron complex for the asymmetric hydrophosphorylation of various aldehydes (Scheme 7). The reaction afforded optically active α-hydroxyphosphonates in excellent yields and with good enantioselectivity.

A key feature of this work was the generation of an unusual dinuclear chiral iron complex (chiral Fe complex, 58), formed *in situ* from FeCl_3_ and ligand (57) in the presence of triethylamine in THF at 55 °C (Scheme 7). This complex was subsequently isolated and employed as a catalyst in the asymmetric synthesis of α-hydroxyphosphonates.

The optimal reaction conditions were found to be 10 mol% of the iron catalyst, 0.5 equivalents of sodium carbonate, and heating at 55 °C in THF. The nature of the iron salt had a significant impact on the enantioselectivity: iron(iii) chloride (FeCl_3_) provided superior enantiomeric excesses compared to other iron salts such as FeCl_2_, Fe(OAc)_2_, or FeBr_3_. Moreover, the addition of sodium carbonate and elevated temperature accelerated the reaction rate.

Under these optimized conditions, a broad scope of aromatic and heteroaromatic aldehydes was efficiently converted to the corresponding α-hydroxyphosphonates, with yields ranging from 78% to 96% and moderate enantioselectivities (50–71% *ee*).

**Scheme 8:**
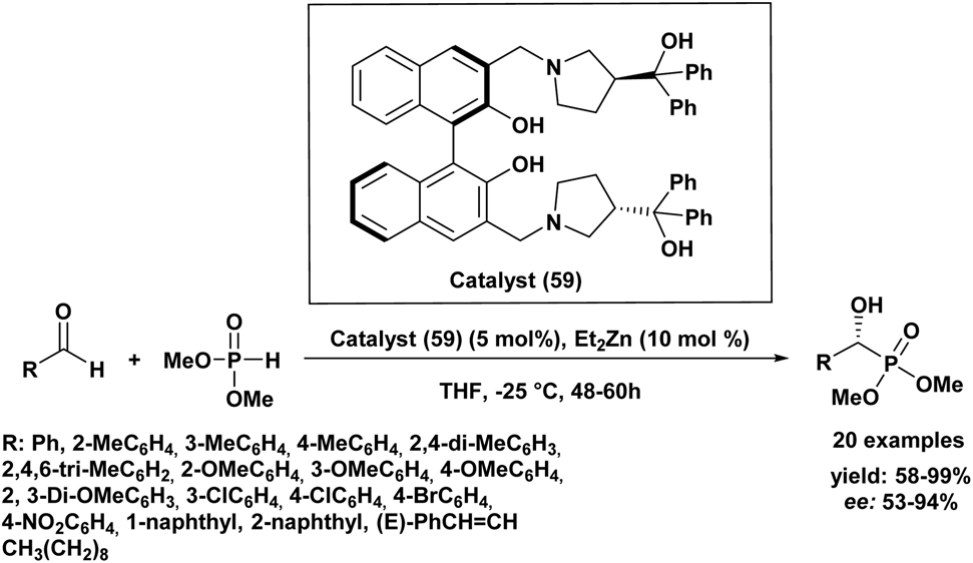
Chiral homobimetallic zinc complex as a catalyst for the asymmetric hydrophosphonylation of aldehydes.

In addition, Chinese researchers^91^ have developed a new method for the asymmetric hydrophosphonylation of aldehydes using a chiral zinc complex as a catalyst (59) (Scheme 8). This catalyst is unique because it has both acidic and basic functional groups, allowing it to activate both the aldehyde and the phosphonate reactant simultaneously. Under specific conditions (5 mol% catalyst (59), 48–60 hours, −20 °C in THF), they successfully converted various aldehydes, mainly substituted benzaldehydes, into chiral products with moderate to excellent yields (58–99%) and enantioselectivities (53–94%). The outcome of the reaction was influenced by both the electronic and steric properties of the aldehydes. For instance, benzaldehydes bearing electron-donating groups at the *meta* position generally afforded better yields and higher enantiomeric excesses, whereas electron-withdrawing groups led to lower enantioselectivities. This work provides a new and useful catalyst for creating enantioenriched α-hydroxyphosphonates.

**Scheme 9:**
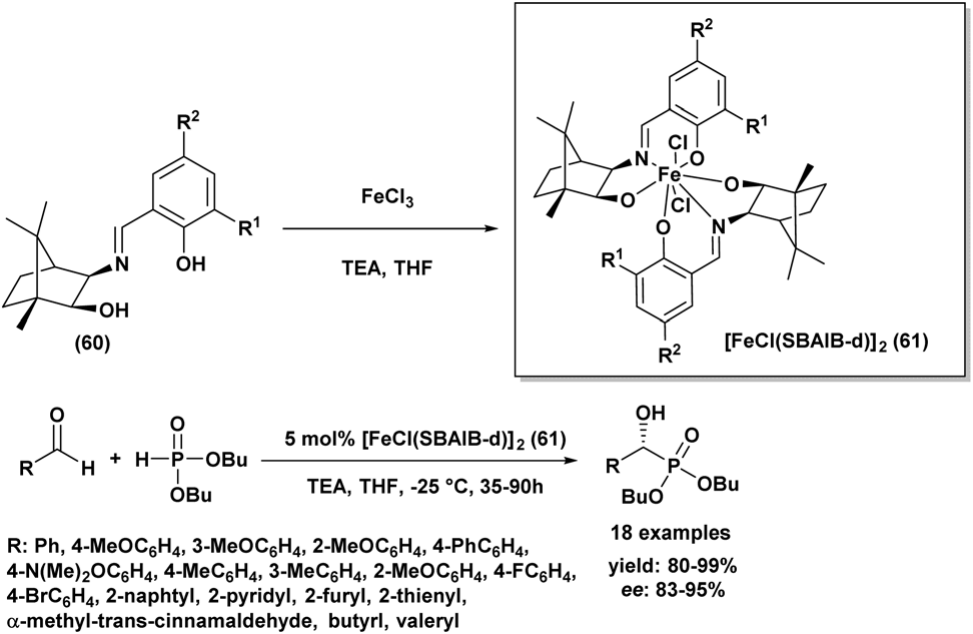
[FeCl(SBAIB-d)]_2_ catalyzed asymmetric hydrophosphonylation of aldehydes.

In 2013, Chen and Boobalan^92^ introduced a highly effective catalytic system for asymmetric hydrophosphonylation, employing a Schiff base iron(iii) complex [FeCl(SBAIB-d)]_2_(61), derived from amino-isoborneol (SBAIB) (60). This catalyst, synthesized in high yield from the reaction of the Schiff base of (+)-aminoisoborneol with ferric chloride and triethylamine in THF (as depicted in Scheme 9), demonstrated that the imine functionality was critical for achieving high enantioselectivity. Through optimization, they found that using 5 mol% of the complex with specific substituents (R_1_ = Me, R_2_ = H) was optimal, leading to excellent enantioselectivities (up to 99% ee) and high yields (up to 99%) across various aldehydes. The catalyst with an *ortho*-methyl substituted phenol ligand provided the best stereocontrol, which was further enhanced by using dibutyl phosphite (1.2 eq.) as a nucleophile to increase steric hindrance. The reactions were conducted at −25 °C in THF with a stoichiometric amount of triethylamine and proved applicable to a wide range of aryl, heteroaryl, alkylidene, and alkyl aldehydes, affording excellent yields (80–99%) and high to excellent enantiomeric excesses (83–95% ee). A screening of seven synthesized Fe(iii)-SBAIB complexes identified [FeCl(SBAIB-d)]_2_ as the most effective, yielding the corresponding diisopropyl α-hydroxy phosphonate with 79% ee in a model reaction and up to 99% *ee* in other instances. This catalytic system offers advantages such as easy synthesis from inexpensive materials, mild reaction conditions, and high enantioselectivities with low catalyst loading.

**Scheme 10:**
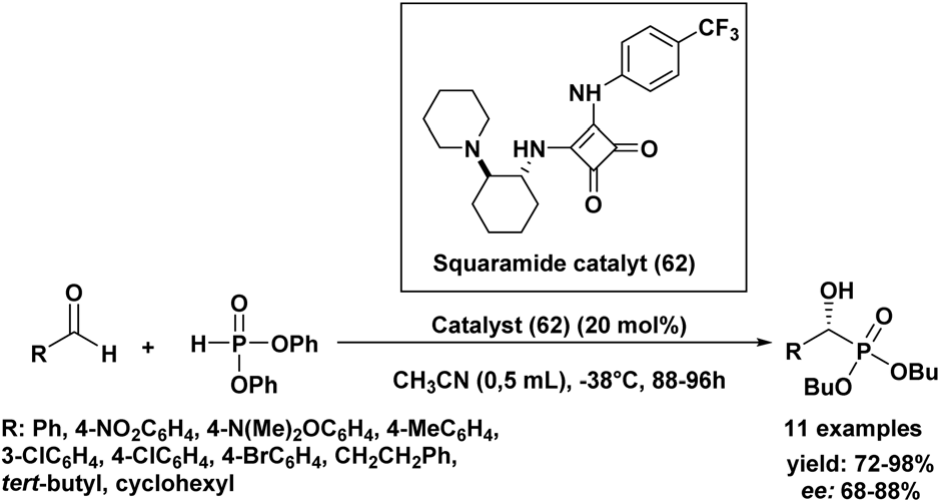
Asymmetric hydrophosphonylation of aldehydes catalyzed by a squaramide-based organocatalyst.

In 2014, interesting results were reported using a squaramide organocatalyst for the asymmetric Pudovik reaction with various aldehydes (Scheme 10).^93^ Building upon this, the optimal reaction conditions determined in our study involve the use of squaramide (62) as the chiral catalyst and diphenyl phosphite as the phosphite source. The reaction is best conducted in acetonitrile (CH_3_CN) as the solvent at a temperature of −38 °C, surprisingly without stirring, and with a catalyst loading of 20 mol%, which provides a reasonable balance between yield and catalyst usage. Under these mild reaction conditions, the addition of phosphite to various aldehydes yielded α-hydroxy phosphonates in very good yields and with high enantiomeric excesses (68% to 88% ee) across the board. Notably, the electronic properties of substituents on aromatic aldehydes showed no discernible effect on the reaction's success, while among aliphatic aldehydes, pivalaldehyde afforded the best chiral induction. This method represents a further expansion of organocatalytic approaches for the hydrophosphonylation of aldehydes, with the significant advantage of utilizing commercially available reagents and catalysts.

**Scheme 11:**
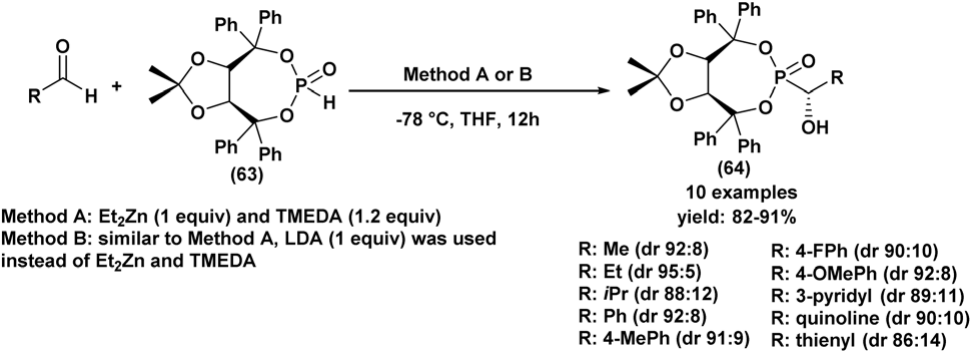
Asymmetric synthesis of α-hydroxyphosphonates using a chiral H-phosphonate.

In 2015, Olszewski^94^ described a diastereoselective hydrophosphonylation of various aldehydes using a chiral auxiliary H-phosphonate (*R*, *R*)-4,5-bis(diphenylhydroxymethyl)-2,2-dimethyl-1,3-dioxolane (69) auxiliary H-phosphonate (Scheme 11).

Optimization studies revealed that the best results were achieved using two closely related procedures. Method (A) involved performing the reaction in THF at −78 °C with diethylzinc (Et_2_Zn) and TMEDA. Method (B) followed a similar approach but employed lithium diisopropylamide (LDA) to generate Et_2_Zn *in situ*, alongside TMEDA. Both methods proved effective in promoting the diastereoselective hydrophosphonylation under mild conditions.

The auxiliary H-phosphonate was prepared from the readily available and commercially accessible (*R*, *R*)-4,5-bis(diphenylhydroxymethyl)-2,2-dimethyl-1,3-dioxolane (TADDOL) by reaction with phosphorus trichloride (PCl_3_) in the presence of triethylamine (TEA) in water. Different aliphatic, aromatic, and heteroaromatic aldehydes were tolerated under the presented, optimized reaction conditions. The yields of the final α-hydroxyphosphonates (64) were good, ranging from 82% to 91% for pure and isolated products. Small variations in the diastereomeric ratio were observed, influenced by the presence of electron-donating or electron-withdrawing groups on the aromatic ring. This approach presents a versatile and efficient method for the diastereoselective synthesis of α-hydroxy methylphosphonates (63), accommodating structurally diverse aldehydes through slight variations in reaction conditions.

**Scheme 12:**
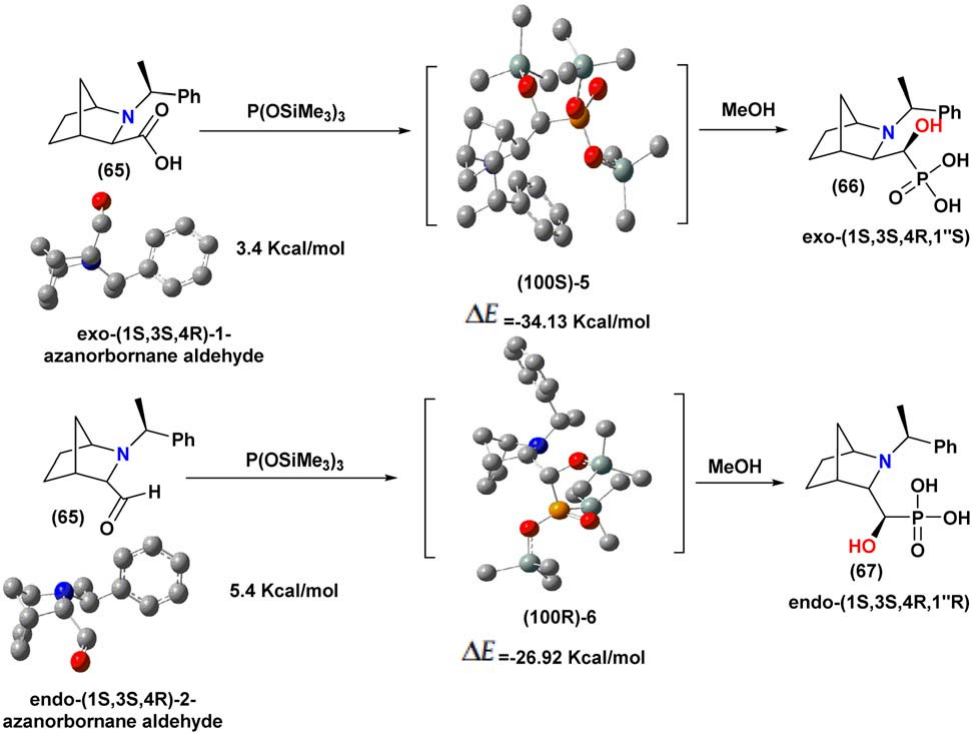
Proposed stereochemical outcome and DFT-calculated structures for the hydrophosphonylation of exo- and endo-2-azanorbornane aldehydes with P(OSiMe_3_)_3_, yielding the thermodynamically favored phosphonate products (100S)-5 and (100R)-6, which upon methanol dealkylation produce α-hydroxyphosphonic acids with conserved stereochemistry. Relative conformer stabilities and reaction free energies (Δ*E*) were obtained at the M06-2X/6-311++G(2d,2p) level.

To gain deeper insight into the stereochemical outcome of this transformation, theoretical studies were also undertaken. The same authors^95^ also investigated the origin of the stereoselectivity observed in hydrophosphonylation reactions using quantum chemical calculations. DFT studies were performed to examine the reaction between azanorbornane aldehydes (65) (exo-1 and endo-2) and P(OSiMe_3_)_3_. For each substrate, two conformers were identified, and the most stable ones were selected for further analysis based on their relative energies (exo-1: 3.4 kcal mol; endo-2: 5.4 kcal mol^−1^) (Scheme 12).

The computational results indicated that hydrophosphonylation preferentially occurs through nucleophilic attack of the phosphorus reagent on the less sterically hindered face of the aldehyde carbonyl, leading to the formation of the thermodynamically favored phosphonate intermediates (100S)-5 and (100R)-6, with reaction free energies of (−34.13 kcal mol^−1^ and −26.92 kcal mol^−1^, respectively. The stereochemical outcome is mainly governed by steric effects imposed by the azanorbornane framework and the *N*-substituent, which block one side of the carbonyl group and direct the nucleophilic attack.

Subsequent methanol dealkylation of these intermediates preserves the stereochemistry at the carbon adjacent to phosphorus, affording the corresponding α-hydroxyphosphonic acids with defined configurations. These theoretical results provide mechanistic insight into the high stereoselectivity of hydrophosphonylation reactions and highlight the key role of steric factors in controlling the reaction pathway.

**Scheme 13:**
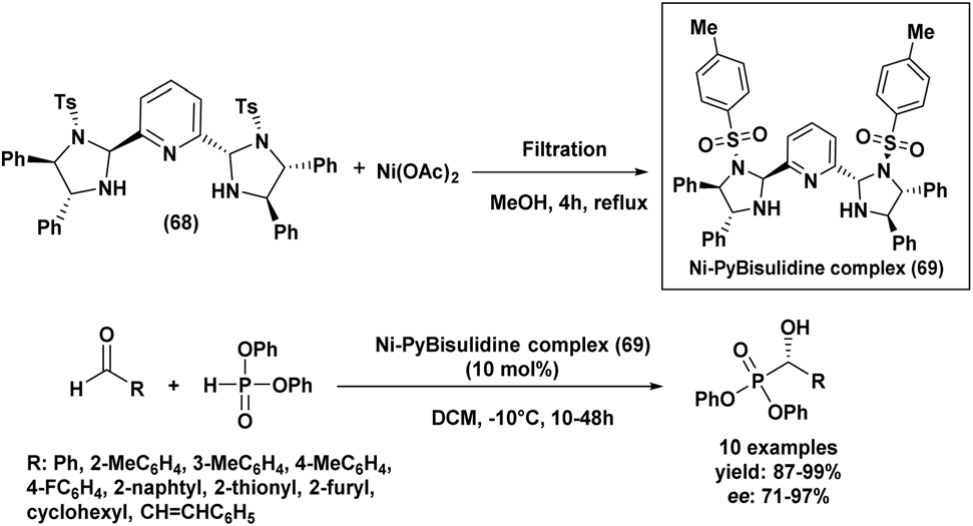
PyBisulidine as ligand for Ni-catalyzed hydrophosphonylation of aldehydes.

The nickel complex Ni–PyBisulidine (69), developed by Zeng and co-workers^96^ in 2016, serves as a highly efficient catalyst for the enantioselective hydrophosphonylation of a broad range of aldehydes (Scheme 13). Generated *in situ* from PyBisulidine (68) and nickel acetate, this catalytic system facilitated the asymmetric addition of diphenylphosphite to various aldehyde substrates, including aromatic, heteroaromatic, cycloalkyl, and α,β-unsaturated derivatives. This method afforded the corresponding α-hydroxy phosphonates in high yields (87–99%) and with good to excellent enantioselectivities (71–97%) (Scheme 13). A significant advantage of this catalytic system is its robustness under air and its compatibility with common solvents like methanol, toluene, and THF. The straightforward procedure is operationally simple and environmentally sound. Moreover, the use of a 1 : 1 ratio of reactants not only boosts efficiency but also improves atom economy in the synthesis of α-hydroxy aromatic phosphonates, making it a compelling tool for sustainable asymmetric synthesis.

**Scheme 14:**
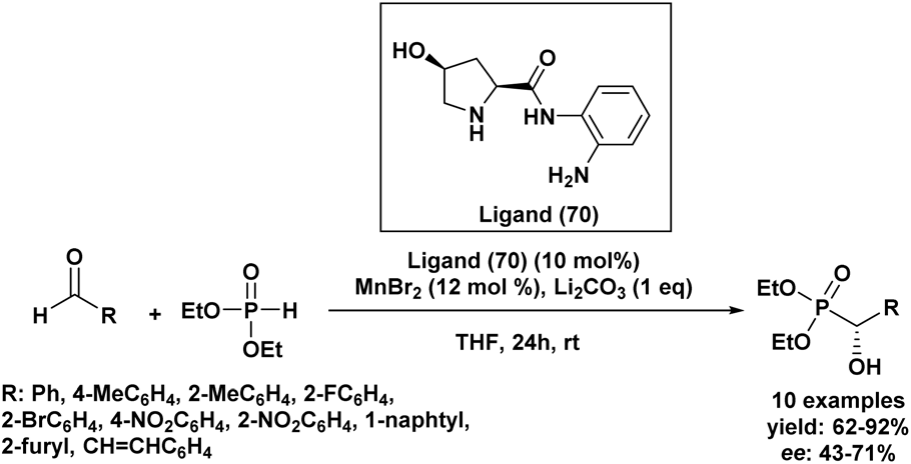
Proline derivative as ligand for Mn-catalyzed hydrophosphonylation of aldehydes.

In 2017, Lim *et al.*^97^ developed an efficient catalytic system for the enantioselective synthesis of aromatic α-hydroxyphosphonates, employing manganese as a Lewis acid and a chiral hydroxyprolinamide ligand. The study focused on the asymmetric hydrophosphonylation of benzaldehyde using diethyl phosphite, lithium carbonate, and a proline-derived chiral ligand (70) in the presence of a manganese salt (Scheme 14). While manganese(ii) chloride initially showed promising results (72% yield, 62% ee), manganese(ii) bromide offered improved performance. A screening of various proline-based ligands identified ligand (70) as the most effective, achieving an enantiomeric excess of 71%, highlighting the critical role of the free amine group in metal coordination. Anhydrous THF and acetonitrile were found to be the most suitable solvents, and lithium carbonate emerged as the optimal base. Under optimized conditions (10 mol% ligand (70), 12 mol% MnBr_2_, 1 equiv of Li_2_CO_3_ in anhydrous THF at room temperature), the methodology was extended to a range of aldehydes.

The nature and position of substituents on the aromatic ring had a pronounced effect on both the yield and enantioselectivity of the reaction. Aromatic aldehydes bearing electron-withdrawing groups, such as nitro, cyano, or halogen substituents, generally led to diminished yields and lower enantiomeric excesses. Steric hindrance from *ortho*-substituents on benzaldehydes hindered the reaction, lowering yield and enantioselectivity, likely by limiting catalyst access. The complete unreactivity of 2-nitrobenzaldehyde shows the severe impact of combined steric and electronic effects at the *ortho* position. Diethyl phosphite was the most effective reagent, yielding higher amounts of the desired product with better enantioselectivity compared to dimethyl or dibutyl phosphites. This superior performance likely stems from its optimal balance of reactivity and steric compatibility with the catalytic system.

### 3.3. Comparative analysis of Abramov and Pudovik methodologies

Although both Abramov and Pudovik reactions provide efficient routes toward α-hydroxyphosphonates, the two methodologies rely on distinct activation modes and therefore exhibit different synthetic features, advantages, and limitations. From a mechanistic perspective, the Abramov reaction involves the nucleophilic addition of trialkyl phosphites to carbonyl compounds, generally promoted by Lewis acids, halogen activators, or Brønsted acids that increase the electrophilicity of the carbonyl group. In contrast, the Pudovik reaction proceeds through base activation of dialkyl phosphites, generating a nucleophilic phosphite anion that attacks the carbonyl substrate. Consequently, the choice between these two approaches is often dictated by the nature of the substrate and the desired reaction conditions.

In terms of substrate scope, both reactions display broad applicability toward aromatic aldehydes, which generally afford the highest yields. However, ketones are typically less reactive in both systems due to steric hindrance and reduced electrophilicity. In Abramov reactions, Lewis acid catalysis has proven particularly effective for activating less reactive ketones, whereas Pudovik reactions often require stronger bases or longer reaction times to achieve comparable conversions. From a sustainability standpoint, recent developments have focused on solvent-free conditions, recyclable heterogeneous catalysts, and alternative activation methods such as ultrasound or microwave irradiation. In Abramov chemistry, supramolecular systems (*e.g.*, β-cyclodextrin) and sonochemical activation have enabled efficient transformations under mild and environmentally benign conditions. Similarly, Pudovik reactions have benefited from heterogeneous basic catalysts such as modified apatites, metal oxides, and eco-derived catalysts, which offer excellent recyclability and reduced environmental impact.

Beyond these mechanistic and practical considerations, important differences also emerge in the field of asymmetric synthesis. Indeed, the asymmetric synthesis of α-hydroxyphosphonates has been extensively explored through the Pudovik reaction, whereas asymmetric variants of the Abramov reaction remain comparatively limited. This disparity can be rationalized by the reaction mechanism. In the Pudovik reaction, the formation of a phosphite anion allows efficient interaction with chiral catalytic environments, thereby facilitating stereochemical control. As a result, numerous catalytic systems have been developed, including chiral Lewis acids, organocatalysts, and metal complexes, often providing good enantioselectivities and broad substrate scope, particularly with aromatic aldehydes.

In contrast, asymmetric Abramov reactions are less developed because the reaction generally proceeds under Lewis acid activation and involves mechanistic pathways that make stereocontrol more challenging. Moreover, several reported methods require specific reagents or relatively harsh conditions, which can limit the efficiency of chiral induction. Consequently, many asymmetric Abramov protocols still provide only moderate enantioselectivities and remain limited in terms of substrate scope.

Overall, the Pudovik reaction currently represents the most efficient and widely used strategy for the asymmetric synthesis of α-hydroxyphosphonates, whereas further methodological developments are still required to expand the potential of asymmetric Abramov reactions.

### 3.4. Alternative synthetic approaches to α-hydroxyphosphonates

The synthesis of α-hydroxy phosphonates can be achieved through various alternative routes beyond the classical Pudovik and Abramov pathways. Aldol-type condensations between phosphonates and carbonyl compounds provide α-hydroxy phosphonates under basic or Lewis acid catalysis. The Michaelis–Arbuzov reaction, involving trialkyl phosphites and α-haloketones, offers another route. Oxidation of *α*-alkyl phosphonates can yield hydroxyphosphonates derivatives, while catalytic hydrogenation of α-keto phosphonates also affords α-hydroxyphosphonates with good stereocontrol. These methods expand synthetic versatility for bioactive compounds development.

Several alternative synthetic routes to α-hydroxy phosphonates were described above, each presenting specific advantages in reactivity, selectivity, and functional group compatibility.

**Scheme 15:**
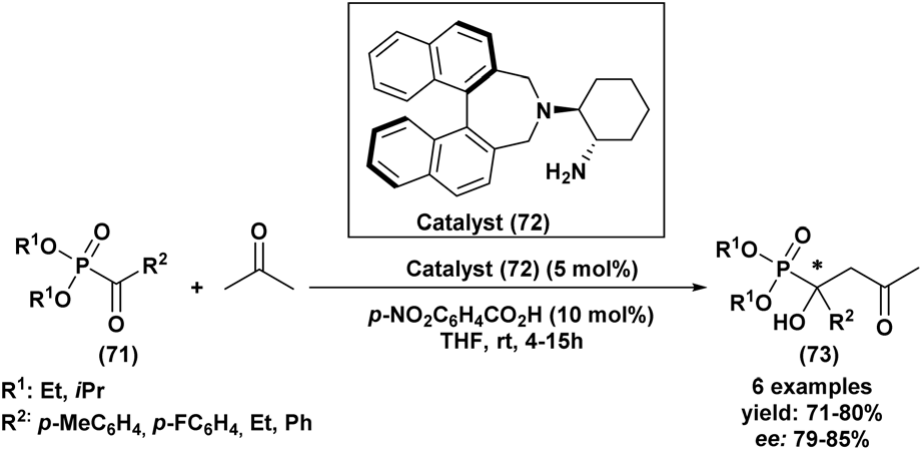
Enantioselective synthesis of tertiary α-hydroxy phosphonates using a 1,2-diaminocyclohexane-binaphthyl as catalyst.

In 2011, Lee *et al.*^98^ introduced a novel organocatalytic method for the enantioselective synthesis of chiral tertiary α-hydroxy phosphonates (73) through an aldol reaction between acetone and α-keto phosphonates (Scheme 15). The optimization of the reaction conditions for this aldol reaction started with the screening of various organocatalysts. A chiral 1,2-diaminocyclohexane-binaphthyl derivative catalyst (72) demonstrated initial potential, yielding the product with 62% yield and 60% enantiomeric excess (ee). Subsequent optimization, focusing on catalyst (72), involved exploring different acidic additives. This led to the identification of 4-nitrobenzoic acid as the most effective additive, enhancing the yield to 78% and the enantioselectivity to 75% ee. Solvent screening indicated that while several common solvents were suitable, tetrahydrofuran (THF) provided a slightly improved enantioselectivity of 79% ee. Notably, the catalyst loading could be reduced to 5 mol% without significantly compromising the yield or enantioselectivity. These optimized conditions proved generally applicable to a range of α-keto phosphonates (71), affording the corresponding chiral tertiary α-hydroxy phosphonates (73) in good to high yields and with enantioselectivities up to 95% *ee*. Further studies on the reaction mechanism and applications were promised in future reports.

**Scheme 16:**
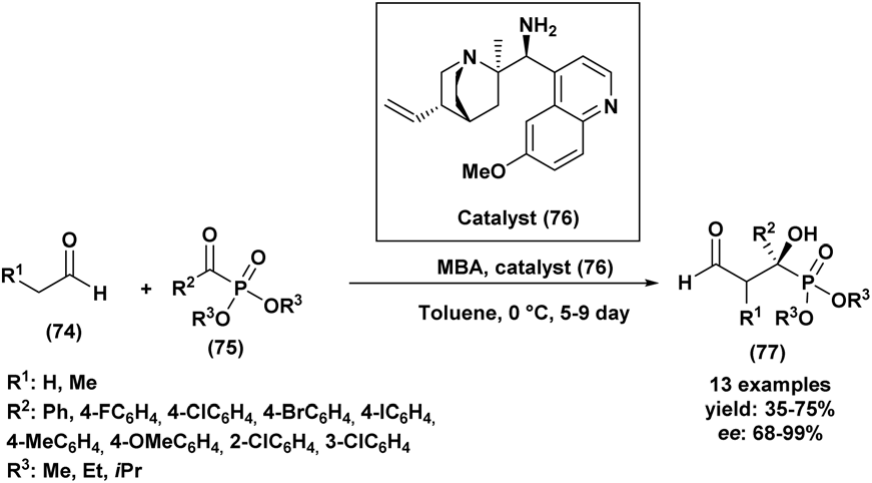
Synthesis of β-formyl-α-hydroxyphosphonates *via* a cross-aldol reaction catalyzed by 9-amino-9-deoxyepiquinine.

An American research team^99^ has achieved a significant milestone in organic chemistry by developing the first highly enantioselective cross-aldol reaction involving enolizable aldehydes and α-ketophosphonates (Scheme 16). This novel method efficiently produces tertiary β-formyl-α-hydroxyphosphonates (77) with exceptional stereocontrol. The reaction is effectively catalyzed by just 10 mol% of 9-amino-9-deoxyepi-quinine (76) and 30 mol% of 4-methoxybenzoic acid, a primary amine derived from quinine. Through optimization, toluene was identified as the optimal solvent, with reactions proceeding smoothly at 0 °C. This process consistently delivered high yields (70–95%) and outstanding enantioselectivities (up to 99% *ee*). Notably, even acetaldehyde, a notoriously difficult substrate due to its high reactivity and self-condensation tendencies, was efficiently converted with excellent enantioselectivity (ee: 93%), a feat previously unachieved with primary amine organocatalysts. The new method demonstrates broad substrate scope and good tolerance for various functional groups. An optimal aldehyde to α-ketophosphonate ratio of 1.2 : 1 was found to maximize both selectivity and conversion.

Beyond its synthetic utility, the resulting β-formyl-α-hydroxyphosphonates (77) showed promising biological activity. Preliminary screening revealed significant anticancer properties, effectively inhibiting the proliferation of human and murine tumor cell lines. Crucially, these compounds exhibited minimal toxicity to normal human fibroblast cells, suggesting selective antiproliferative effects and significant potential for developing new therapeutic agents.

**Scheme 17:**
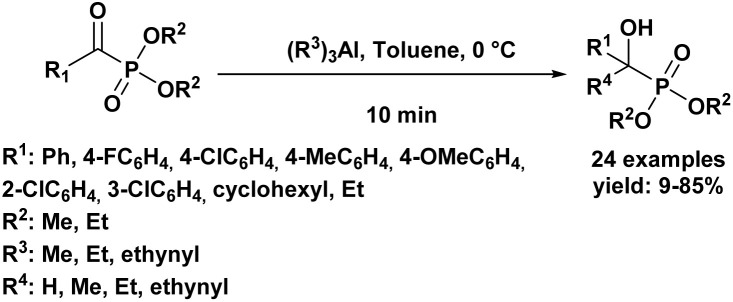
Secondary and tertiary α-hydroxyphosphonates synthesized through 1,2-addition of organoaluminum reagents to acyl phosphonates.

Another study reported the synthesis of 24 secondary and tertiary α-hydroxyphosphonates through the 1,2-addition of commercially available trimethylaluminum to a series of substituted acyl phosphonates (Scheme 17),^100^ prepared *via* the Michaelis–Arbuzov reaction. Various solvents, including THF, toluene, dichloromethane, and hexane, were screened at 0 °C to evaluate their influence on the 1,2-addition of trimethylaluminum to benzoyl phosphonate. Among these, toluene gave the highest yield of α-hydroxyphosphonate, likely due to its optimal balance of coordinating ability and solubility, which facilitates the dissociation of dimeric aluminum species and enhances the reactivity of the reagent. Consequently, toluene was selected as the solvent of choice for all subsequent reactions. Optimal yields of α-hydroxyphosphonates were achieved using 3 equivalents of trimethylaluminum (Me_3_Al), as lower amounts led to incomplete conversion. The nature of substituents on the benzoyl phosphonates influenced reactivity: electron-withdrawing groups (*e.g.*, Cl, F) improved yields, while electron-donating groups like Me and OMe reduced them. Reaction temperature determined product type: secondary α-hydroxyphosphonates were formed at 0 °C *via* hydride addition, whereas tertiary products resulted from alkyl transfer at −100 °C, though in lower yields. Similar behavior was observed with Et_3_Al and Al(*i*-Bu)_3_, both favoring hydride addition over alkylation under mild conditions. Alkynylation using triethynylaluminum also afforded α-hydroxyphosphonates in moderate yields after optimization of reaction conditions.

**Scheme 18:**
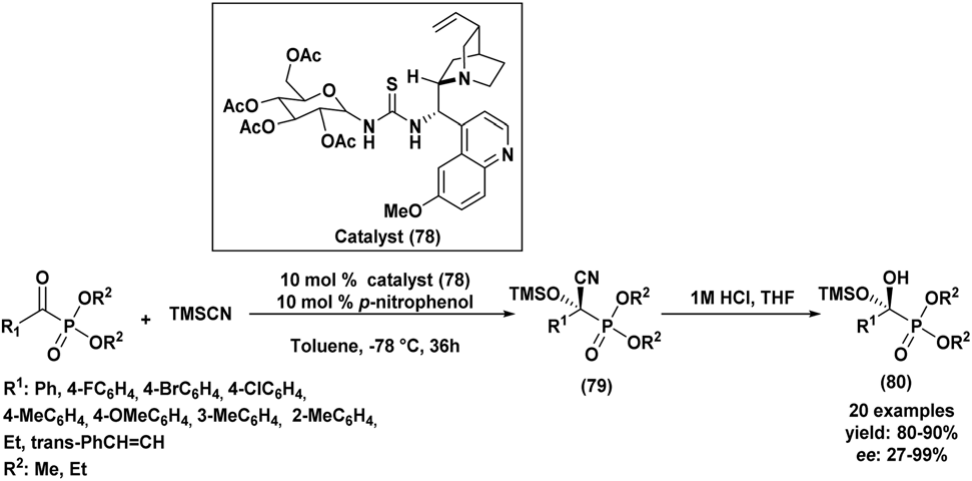
Organocatalytic asymmetric cyanation of α-ketophosphonates using cinchona/carbohydrate-based thioureas.

Kong and co-workers^101^ developed an efficient and highly enantioselective two-step strategy for the synthesis of tertiary α-hydroxyphosphonates. The method involves the asymmetric addition of trimethylsilyl cyanide (TMSCN) to α-ketophosphonates, followed by mild acidic hydrolysis (Scheme 18). Through catalyst screening, the thiourea catalyst featuring both cinchona alkaloid and carbohydrate moieties (78) proved to be the most effective in promoting the first step with excellent stereocontrol. Under optimized conditions including 10 mol% of catalyst, 10 mol% of *p*-nitrophenol as an additive, and toluene as the solvent at −78 °C for 36 hours the reaction delivered excellent results, achieving up to 90% yield and an outstanding 99% enantiomeric excess (*ee*). This method exhibited a broad substrate scope, efficiently transforming a variety of aromatic α-ketophosphonates into the corresponding α-hydroxyphosphonates (80) with high enantioselectivities, typically ranging from 83% to 99% ee. Notably, substrates bearing substituents at the *ortho* position showed a marked drop in enantioselectivity, with the *ee* decreasing to 58%. Additionally, increased steric bulk on the substrate or the use of aliphatic α-ketophosphonates resulted in reduced enantioselectivity. Nonetheless, the method successfully accommodated α,β-unsaturated cinnamoyl phosphonates, achieving high yield and *ee* without any detectable side products, underscoring the selectivity and robustness of the catalytic system. The absolute configuration of the main product was determined as (*S*) by single crystal X-ray diffraction. Mechanistic studies using ^31^P NMR revealed that the thiourea catalyst forms a hydrogen-bonded complex with the ketophosphonate, while *p*-nitrophenol facilitates cyanide generation. The catalyst's cinchona and carbohydrate units create a chiral environment that promotes selective attack from the Si face, enabling high stereo control.

**Scheme 19:**
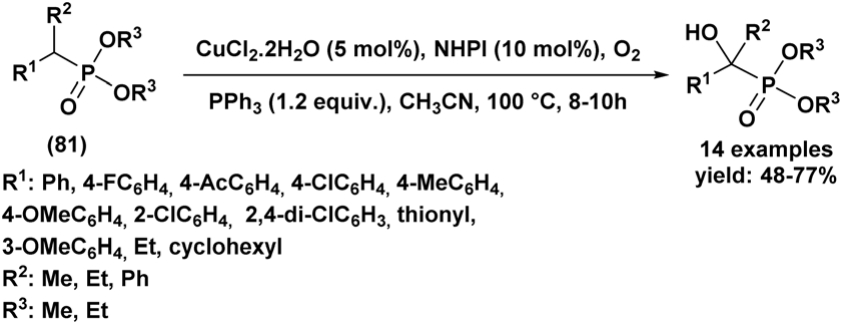
Quaternary α-hydroxyphosphonates synthesized *via* copper-catalyzed aerobic hydroxylation of phosphonates.

In a 2015 publication, Gu *et al.*^102^ reported a copper-catalyzed aerobic oxidation method for the synthesis of quaternary α-hydroxyphosphonates from phosphonate derivatives (Scheme 19). Using dimethyl-1-phenylethylphosphonate (81) as the model substrate, the optimized conditions comprising CuCl_2_·2H_2_O (5 mol%), NHPI (10 mol%), PPh_3_ (1.2 equiv.), in acetonitrile under an O_2_ atmosphere at 100 °C afforded the desired product in up to 77% yield for 8–10 h. Key control experiments confirmed the crucial roles of PPh_3_, O_2_, and CuCl_2_, with radical inhibition by TEMPO and ^18^O-labeling studies suggesting a radical mechanism involving molecular oxygen as the oxygen source.

The methodology demonstrated broad substrate compatibility, delivering well to high yields (48–77%) with both aryl- and alkyl-substituted phosphonates. Functional group tolerance was confirmed with electron-withdrawing and electron-donating aryl substituents, heteroaryl analogues, and even aliphatic phosphonates. Mechanistic investigations supported a pathway involving formation of a phthalimide *N*-oxyl (PINO) radical, hydrogen abstraction from the substrate, O_2_ trapping, and final reduction of a hydroperoxide intermediate by PPh_3_ to yield the α-hydroxyphosphonate.

The same research group^103^ found another approach for C–H hydroxylation of phosphonates using molecular oxygen in [bmIm]OH to produce quaternary α-hydroxy phosphonates. This metal-free method proceeds under mild conditions at room temperature and atmospheric pressure, using triethyl phosphite as a reductant. The reaction showed good efficiency, affording the desired products in moderate to high yields. Wide ranges of aromatic and heteroaromatic phosphonates were tolerated, and functional groups such as methoxy, halogens, and multiple substitutions had no significant negative effect on the outcome. Mechanistic studies confirmed that molecular oxygen is the source of the hydroxyl group and that the transformation likely proceeds *via* a non-radical pathway. This greener and practical protocol offers a valuable alternative to existing hydroxylation methods.

**Scheme 20:**
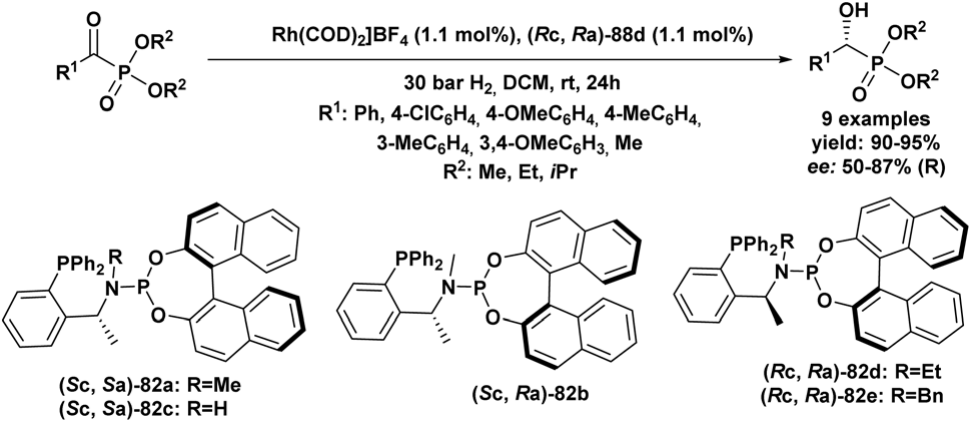
Rhodium-catalyzed asymmetric reduction of challenging α-keto phosphonates.

Li *et al.*^104^ reported a Rh-catalyzed asymmetric hydrogenation of sterically hindered α-keto phosphonates using a newly developed chiral phosphine-phosphoramidite ligand (Scheme 20). To optimize enantioselectivity and yield, various ligands were evaluated. Among them, ligand (*S*c, *S*a)-82a delivered the best result with a 92% yield and 81% enantiomeric excess (ee), whereas ligands such as (*S*c, *R*a)-82b and (*S*c, *S*a)-82c gave lower selectivities, underscoring the critical influence of ligand stereochemistry and steric environment. To enhance stereocontrol, new ligands (*R*c, *R*a)-82d and 82e were synthesized from (*R*)-α-phenylethylamine. Ligand 82d exhibited good enantioselectivity (87% ee), while 88e proved less effective. Solvent screening identified CH_2_Cl_2_ as the optimal medium, whereas solvents like toluene, THF, MeOH, and EtOAc gave poor results. Under the optimized conditions which involved 1.1 mol% Rh(COD)_2_]BF_4_ and 1.1 mol% (*R*c, *R*a)-82d at room temperature under 30 bar H_2_ for 24 hours (Scheme 20), a broad range of α-keto phosphonates underwent successful asymmetric hydrogenation. Aryl α-keto phosphonates bearing electron donating (methyl, methoxy) and electron-withdrawing (chloro) groups at the *meta*- and *para*-positions of the phenyl ring gave good yields and moderate to good enantioselectivities, with substituent effects being relatively minor. In contrast, alkyl α-keto phosphonates exhibited significantly lower enantioselectivity (as low as 50% ee, when R_1_ and R_2_ = Me). Moreover, increasing the steric bulk of the ester group (isopropyl ester) led to a marked decrease in enantioselectivity. These results highlight the high efficiency of the Rh/(*R*c, *R*a)-82d catalytic system for a range of aryl substrates, while also revealing its sensitivity to steric hindrance in both the substrate and ester moiety, and its reduced performance with aliphatic substrates.

**Scheme 21:**
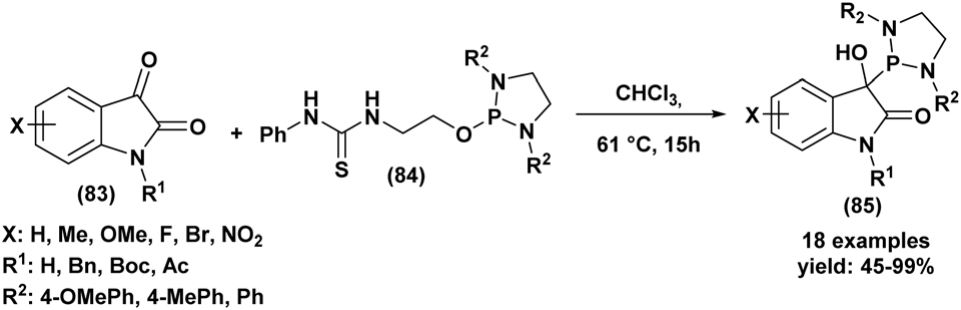
Phospha-aldol approach to prepare α^1^-oxindole-α-hydroxyphosphonates.

In addition, Molleti and Kang^105^ developed an efficient, additive-free methodology for the synthesis of α^1^-oxindole-α-hydroxyphosphonates *via* a phospha–aldol reaction between isatins (83) and bifunctional NHP-thioureas (84) (Scheme 21). This transformation relies on the synergistic hydrogen-bond activation of the carbonyl group by the thiourea moiety, which facilitates the nucleophilic attack of a phosphine. Following systematic optimization, they identified ideal conditions: a slight excess of NHP-thiourea (1.5 equiv.), chloroform as solvent, and a reaction temperature of 61 °C, affording excellent yields of up to 99% for 15 h. Under these conditions, the substrate scope study revealed that a wide range of isatin derivatives could undergo the phospha–aldol reaction efficiently. Isatins bearing either electron-donating or electron-withdrawing groups on the aromatic ring gave good to excellent yields, highlighting the method's broad functional group tolerance. However, steric hindrance from bulky substituents, particularly in *ortho* positions, diminished reactivity. Additionally, the electronic nature of the nitrogen substituent proved critical: *N*-alkyl substituents like methyl and benzyl promoted high yields, whereas electron-withdrawing groups such as Boc or Ac led to reduced efficiency. A plausible mechanism was proposed, involving the formation of a diazaphosphonium intermediate, followed by proton transfer and an intramolecular nucleophilic substitution, ultimately yielding the desired product and thiazolidine as a by-product. This work offers a novel and selective approach to the synthesis of phosphorus-containing oxindole derivatives.

**Scheme 22:**
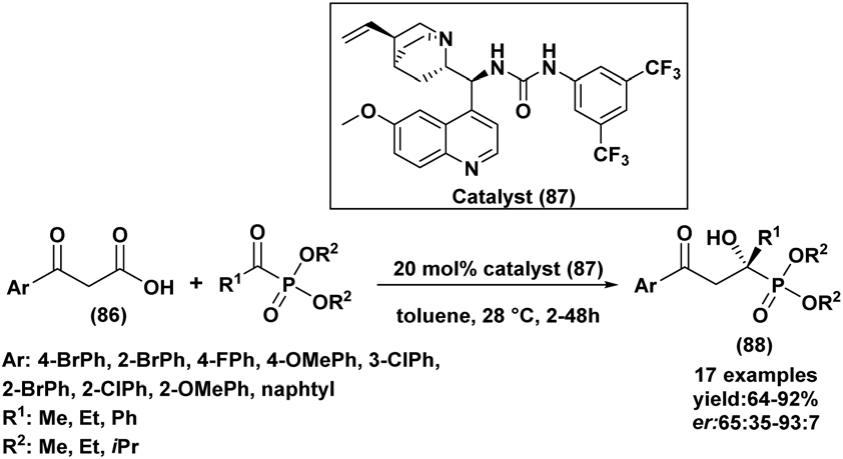
Asymmetric organocatalytic aldol reaction of β-ketoacids with α-ketophosphonates *via* decarboxylation.

In 2017, Vamisetti research group^106^ made a notable contribution to organic chemistry with a publication in Organic & Biomolecular Chemistry. They reported a novel organocatalytic, enantioselective decarboxylative aldol reaction between β-ketoacids (86) and α-ketophosphonates (Scheme 22). This innovative approach enabled the synthesis of chiral γ-carbonyl tertiary α-hydroxyphosphonates (88) bearing a quaternary stereocenter, a key structural motif in many biologically active compounds. Through careful optimization, toluene was identified as the optimal solvent and catalyst (87) (quinidine-derived) provided the best performance. Reducing the catalyst loading to 20 mol% had little effect on enantioselectivity and only slightly lowered the yield. Attempts to further enhance the reaction *via* solvent changes, dilution, additives, or slow addition proved unsuccessful. Under these optimized conditions, the group explored substrate scope, revealing key structure–activity relationships. Larger R_1_ groups on α-ketophosphonates reduced enantioselectivity, while bulkier R_2_ groups decreased reactivity but improved selectivity. Notably, β-ketoacids with *ortho*-substituted aryl groups gave the best results, with enantioselectivity reaching up to 93 : 7 er. Mechanistic insights were gained through ^31^P NMR spectroscopy, which revealed the formation of reaction intermediates. These findings support a stepwise mechanism involving the initial addition of the enolate from the β-ketoacid to the α-ketophosphonate, followed by decarboxylation. The absolute configuration of one product was confirmed *via* single-crystal X-ray diffraction, validating the proposed stereochemical outcome.

**Scheme 23:**
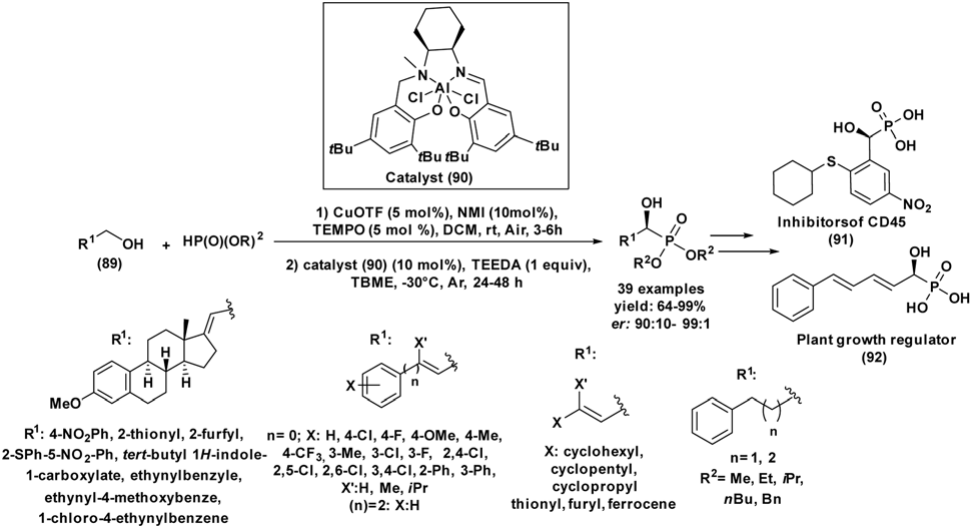
C–H Oxidation and enantioselective phosphonylation of alcohols toward α-hydroxyphosphonates.

In 2021, Ying and co-workers^107^ developed an efficient and versatile protocol for the sequential C–H oxidation and asymmetric phosphonylation of primary alcohols, offering a practical route to access chiral α-hydroxyphosphonates (Scheme 23). The method involves a two-step one-pot process: initially, the alcohol is oxidized to an aldehyde using a Cu(i)/TEMPO/NMI system under an air atmosphere. Subsequently, the *in situ*-generated aldehyde undergoes enantioselective phosphonylation, catalyzed by a chiral aluminum (salalen) complex (90). This tandem approach eliminates the need to isolate intermediates and ensures high efficiency.

The reaction conditions proved compatible with a wide range of alcohol substrates, including benzylic, aliphatic, allylic, and propargylic alcohols, as well as alcohols bearing heterocycles containing sulfur, oxygen, or nitrogen atoms. Across the substrate scope, the method afforded the corresponding chiral α-hydroxyphosphonates in good to excellent yields (64–99%) with high enantioselectivities, demonstrating the broad applicability of the protocol.

Optimization experiments revealed that CuOTf was the most effective copper source for the oxidation step, providing the best enantioselectivity. When evaluating the role of bases, the team found that TEEDA (tetraethylethylenediamine) offered superior results. In contrast, using other bases such as Li_2_CO_3_, K_2_CO_3_, Na_2_CO_3_, TEA, TMEDA, or omitting the base led to a marked decrease in enantioselectivity. Additionally, lowering the reaction temperature from room temperature to −30 °C improved the enantiomeric ratio, achieving up to 99 : 1 er, under the optimized conditions.

This method offers significant advantages due to its reliance on a simple catalytic system and the use of starting materials that are both easily accessible and commercially available. Furthermore, this efficient protocol provides a straightforward route for the synthesis of important compounds, including CD45 (91) inhibitors and plant growth regulators (92), making it a valuable tool in organic synthesis.

### 3.5. Computational studies on α-hydroxyphosphonates

#### 3.5.1. Electronic structure and reactivity analysis

Density Functional Theory (DFT) calculations have become valuable tools for investigating the structural, electronic, and thermodynamic properties of organic compounds.^108,109^ In the case of α-hydroxyphosphonates, these computational approaches provide important insights into molecular stability, charge distribution, frontier molecular orbitals, and reactive sites, thus complementing experimental observations.

A DFT study reported by Uppel *et al.*^110^ investigated diethyl (hydroxy(4-methoxyphenyl)methyl)phosphonate (93) at the B3LYP/6-311G(d,p) level using the Gaussian 09 program. The study focused on molecular geometry optimization, vibrational frequency analysis, frontier molecular orbitals, and thermodynamic properties to better understand the stability and reactivity of the compound. The optimized geometry confirmed the structural stability of the molecule, while Mulliken and Natural Bond Orbital (NBO) analyses revealed that oxygen atoms carry significant negative charges, identifying them as potential nucleophilic sites, whereas the phosphorus atom showed a positive character.

**Fig. 11:**
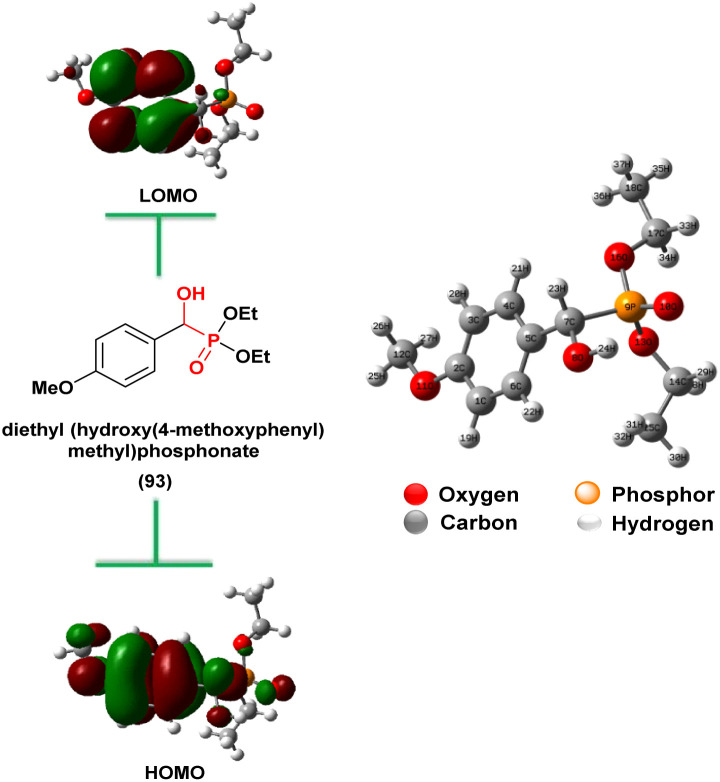
Optimized geometries and 3D distribution of HOMO and LUMO orbitals of studied compound.

Frontier molecular orbital analysis showed a relatively large HOMO–LUMO energy gap (Fig. 11), suggesting good kinetic stability and low chemical reactivity. Vibrational frequency calculations confirmed that the optimized structure corresponds to a true minimum on the potential energy surface, as indicated by the absence of imaginary frequencies. Furthermore, simulated IR and Raman spectra showed good agreement with experimental data after applying appropriate scaling factors.

Thermodynamic parameters including entropy, enthalpy, and heat capacity were also calculated over a range of temperatures, demonstrating the thermal stability of the compound. Experimental thermogravimetric analysis further supported these findings, indicating stability up to about 172 °C before decomposition. Overall, these results highlighted the important role of the phosphonate moiety in governing the electronic distribution and stability of α-hydroxyphosphonates.

Similar theoretical investigations were also carried out on other α-hydroxyphosphonate derivatives to further understand their electronic behavior. In 2019, an Algerian team^111^ examined diethyl [hydroxy(phenyl)methyl]phosphonate and the corresponding phosphonic acid derivative using DFT calculations at the B3LYP/6-31G(d,p) level. The calculated HOMO and LUMO energies, dipole moments, and energy gaps provided useful descriptors for evaluating molecular stability and reactivity.

**Fig. 12:**
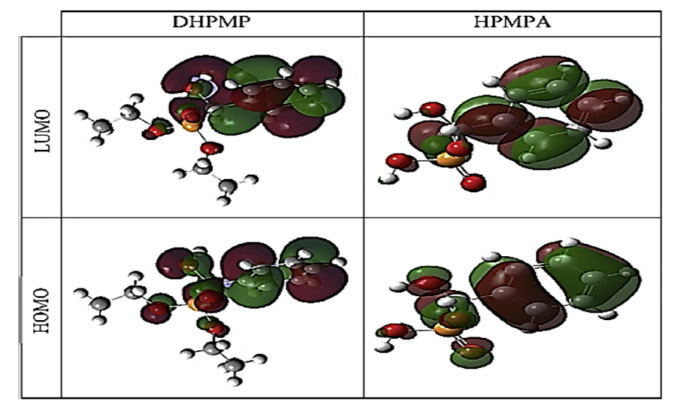
Atomic orbital composition: Positive phase (green) and Negative phase (red).

The study comprehensively investigated the frontier molecular orbitals and adsorption behavior of ester and acid molecules on XC48 steel to elucidate their inhibition performance. Frontier molecular orbital analysis revealed that the highest occupied molecular orbital (E_HOMO_) reflects the electron-donating ability, while the lowest unoccupied molecular orbital (E_LUMO_) indicates the electron-accepting capability of these molecules. The electron density was predominantly localized on the benzene ring, identifying it as the main adsorption site on the metal surface. Both ester and acid derivatives showed relatively small energy gaps (5.59 eV for ester and 5.44 eV for acid), suggesting strong interactions with iron and stable complex formation (Fig. 12).

Moreover, the acid exhibited a higher dipole moment compared to the ester, indicating greater molecular polarity and thus enhanced adsorption onto the steel surface. Their low hardness and high softness values correlated with superior inhibition efficiency, while the global electrophilicity index (*ω*) and fraction of electron transfer (Δ*N* < 3.6) confirmed their effective electron transfer to the metal surface, reinforcing their protective behavior.

**Fig. 13:**
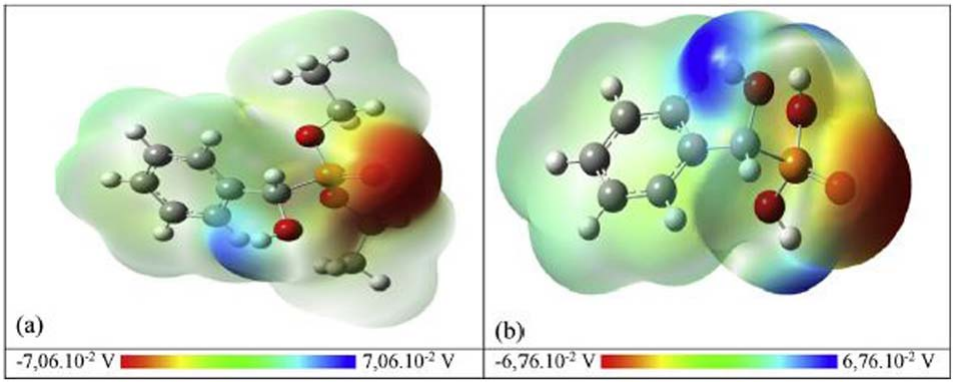
The total electron density mapped with electrostatic potential (a) DHPMP (b) AHPMP.

Natural Bond Orbital (NBO) charge analysis identified negatively charged oxygen and carbon atoms as favorable adsorption sites, whereas the positively charged phosphorus atom may facilitate desorption processes. Molecular Electrostatic Potential (MEP) mapping highlighted nucleophilic regions mainly around the P=O oxygen atom and electrophilic regions near the O–H hydrogens (Fig. 13).

Furthermore, molecular dynamics simulations validated these findings by demonstrating that both molecules adsorb more effectively on Fe (100) surfaces, lying parallel to maximize surface coverage, while adopting a perpendicular orientation on Fe_2_O_3_ (110). Their adsorption involved chemical interactions through coordinate bonds formed by phosphorus and oxygen donating electrons to iron, and dative bonds *via* aromatic π-antibonding orbitals accepting electrons, in addition to physical van der Waals forces. Overall, the higher binding energy and stronger adsorption of the acid molecule confirmed its superior corrosion inhibition performance compared to the ester, in agreement with experimental observations.

**Fig. 14:**
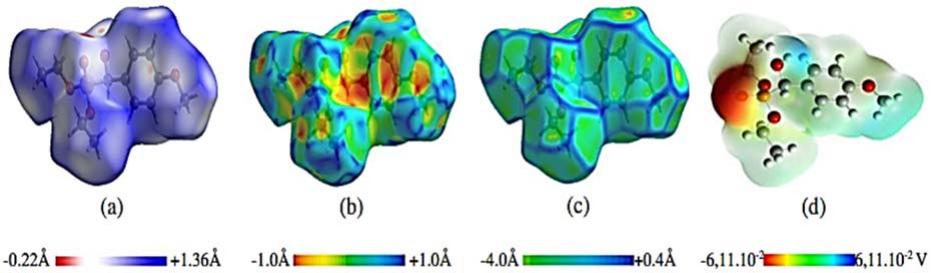
NBO charge analysis for the synthesized α-Hydroxyphosphonate acid ester (DH4MPMP).

In 2020, the same research group^112^ conducted further theoretical studies to explore the properties of their synthesized α-hydroxyphosphonate acid ester (DH4MPMP). DFT calculations were performed to determine second-order nonlinear optical properties based on the first static hyperpolarizability (*β*), revealing that the material may possess significant nonlinear optical behavior. Additionally, frontier molecular orbitals and their energies were calculated to assess electronic reactivity and stability. The research also included the NBO charge analysis and molecular electrostatic potential (MEP) mapping, which showed regions of negative potential around oxygen atoms and positive potential near hydrogen atoms, indicating potential reactive sites. The NBO charge analysis (Fig. 14) showed that the distribution of charges in both the monomer and dimer models is generally similar. All hydrogen atoms carry a net positive charge, with the highest positive charge (1.13e–1.16e) localized on the phosphorus atom. While some carbon atoms exhibit positive charges, others have negative charges. The oxygen atoms display the maximum negative charges, ranging between −0.56e and −0.59e.

Finally, thermodynamic functions such as entropy, heat capacity, and enthalpy were determined from spectroscopic data using statistical methods over the temperature range of 100–1000 K, and thermogravimetric analysis (TGA) was conducted to evaluate the thermal decomposition behavior and stability of the compounds. Thermal analysis estimated stability up to 135 °C, with the *para*-OCH_3_ substituent enhancing thermal resistance, as evidenced by 50% mass loss occurring at 336 °C.

#### 3.5.2. Mechanistic DFT studies

In addition to electronic structure analysis, DFT calculations have also been widely used to elucidate the reaction mechanisms involved in the synthesis of α-hydroxyphosphonates.

For example, Rádai *et al.*^113^ conducted a theoretical investigation on the synthesis of α-hydroxyphosphonates and their α-aminophosphonate derivatives, two important classes of biologically active organophosphorus compounds. Using DFT calculations at the B3LYP/6-31G(d,p) level combined with the PCM solvent model, they analysed the reaction mechanisms and energetics of two key steps: the Pudovik reaction, which affords α-hydroxyphosphonates from carbonyl compounds, and the subsequent nucleophilic substitution with primary amines leading to α-aminophosphonates. In the present discussion, particular attention is given to the formation of α-hydroxyphosphonates *via* the Pudovik reaction.

**Scheme 24:**
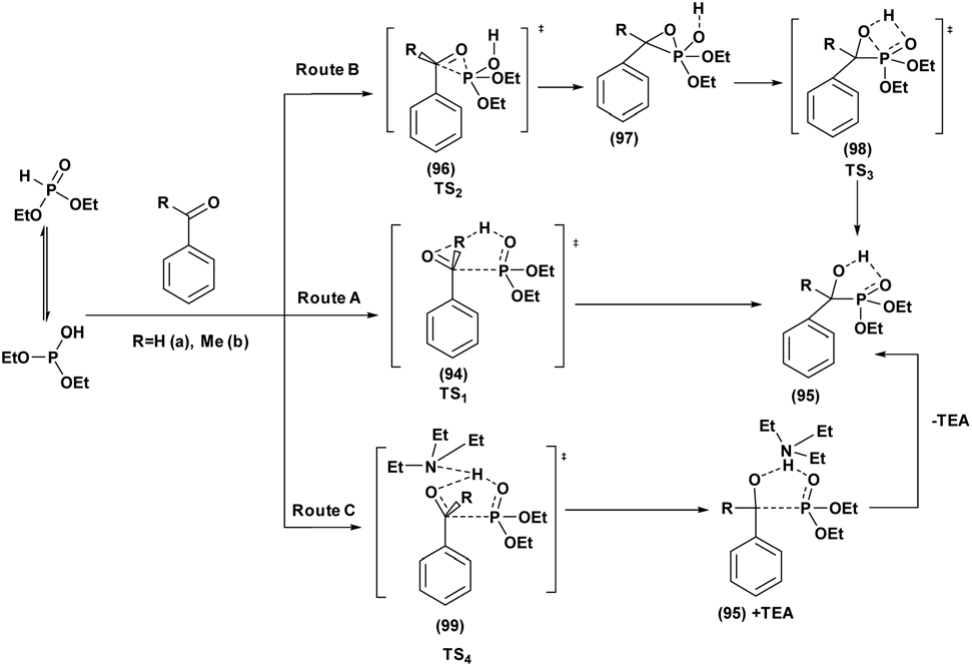
The three proposed mechanistic pathways (routes A-C) for the synthesis of α-hydroxyphosphonates.

The Pudovik reaction may proceed through three distinct mechanistic pathways (Scheme 24). Route A, a one-step direct nucleophilic addition; Route B, a two-step process involving the formation of an epoxyphosphonate intermediate; and Route C, a base-assisted pathway in which triethylamine facilitates proton transfer without complete deprotonation of diethyl phosphite.

**Fig. 15:**
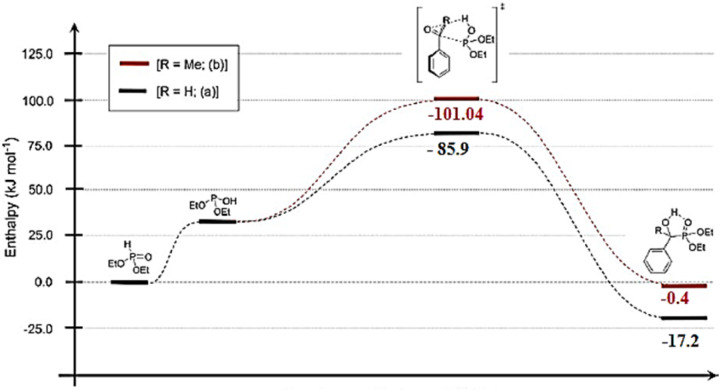
DFT-computed route A for hydroxyphosphonate formation from benzaldehyde and acetophenone precursors.

The results indicate that the Pudovik reaction *via* Route A (Fig. 15) with benzaldehyde proceeds with an activation enthalpy of 85.9 kJ mol^−1^ and is exothermic (−17.2 kJ mol^−1^), suggesting favorable thermodynamics. In contrast, for acetophenone, the activation enthalpy increases to 101.4 kJ mol^−1^ and the reaction becomes nearly thermoneutral (−0.4 kJ mol^−1^). This behavior can be attributed to the presence of the α-methyl group, which introduces steric hindrance and decreases the electrophilicity of the carbonyl group.

The alternative two-step pathway involving an epoxyphosphonate intermediate was also investigated but found to be less favorable due to its higher enthalpy and significant entropy loss. Furthermore, triethylamine does not directly deprotonate diethyl phosphite but rather facilitates proton transfer, lowering the transition-state enthalpy by approximately 18 kJ mol^−1^ and thus accelerating the reaction. Overall, these theoretical results demonstrate that steric and electronic factors strongly influence the efficiency of the Pudovik reaction, with benzaldehyde derivatives being more reactive than acetophenone analogues.

These mechanistic insights contribute to a better understanding of the factors controlling the Pudovik reaction and may help in optimizing reaction conditions for the efficient synthesis of α-hydroxyphosphonates.

## 4. Reactivity of α-hydroxyphosphonates

The distinctive reactivity of α-hydroxyphosphonates arises from the simultaneous presence of a hydroxyl group and a phosphonate moiety on the α-carbon. This unique structural arrangement enables them to undergo a wide range of chemical transformations. Below is a summary of some notable examples of their reactivity.

The 2013 paper by Pallikonda *et al.*^114^ highlights the synthesis of α-aryl/methyl sulfonamidomethyl phosphonates (101) and γ-aryl/methyl sulfonamidomethylvinyl phosphonates (103) from α-hydroxyphosphonates and vinyl hydroxyphosphonates (102), respectively, demonstrating these compounds potential for applications in medicinal and materials science. Hydroxyphosphonates are critical to this synthesis, acting as key substrates in a novel reaction pathway that bypasses the need for complex, moisture-sensitive intermediates and metal catalysts.

Using TfOH as a catalyst, the hydroxyphosphonates facilitate efficient carbocation generation, which in turn drives the sulfonamidation process with compound (100). This method yields high to excellent results under mild, open-air conditions. The study showed that TfOH effectively promotes carbocation formation from α-hydroxyphosphonates, with electron-donating aryl substituents stabilizing the intermediate.

In reactions involving α-hydroxy allylicphosphonates, carbocation stability is enhanced by adjacent double bonds, leading to selective γ-product formation (103). However, when the γ-carbon contains methyl substituents, the reaction instead proceeds *via* an elimination mechanism, yielding a 1,3-butadiene compound.

Additionally, the ability of the hydroxyphosphonates to stabilize carbocations allows for both α- and γ-functionalizations depending on the substituent, offering versatility in synthesizing the sulfamidophosphonate with high selectivity and good yields.

**Scheme 25:**
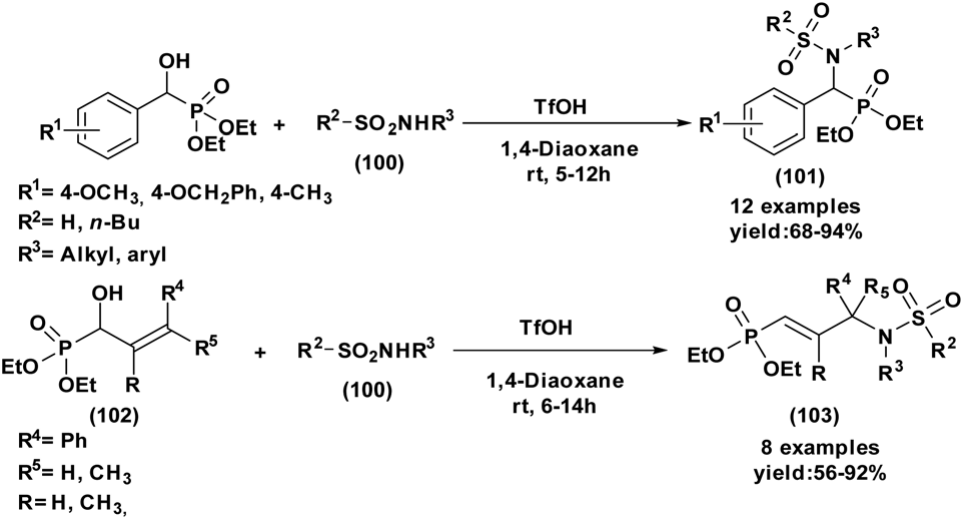
Synthesis of sulfonamidophosphonate starting from α-hydroxyphosphonates.

This metal-free approach also provides environmental and economic advantages by eliminating the need for toxic metal catalysts, minimizing by-products, and using dioxane as a green solvent at room temperature making hydroxyphosphonates promising intermediates for sustainable synthesis in these applications (Scheme 25).

The same group^115^ developed an efficient synthesis of γ-ketophosphonates (105) and (106) through a Lewis acid-catalyzed reaction between α-hydroxyphosphonates and 1,3-diketones (104). The reaction utilizes various Lewis acids, including FeCl_3_, Cu(OTf)_2_, and Bi(OTf)_3_, demonstrating unique regioselective C–C bond cleavage in the 1,3-diketone substrates. Among the catalysts tested, FeCl_3_ and its hydrated form (FeCl_3_·6H_2_O) yielded the best results, particularly under solvent-free conditions, leading to high yields of γ-ketophosphonates (105) and (106).

In investigating optimal conditions, the study revealed that trace amounts of water significantly influenced the reaction outcome. The use of anhydrous FeCl_3_ with a small quantity of water facilitated regioselective C–C bond cleavage in 1,3-diketones, resulting in γ-ketophosphonates as the primary products. The presence of water is thought to stabilize the carbocation intermediate formed during the reaction, enhancing the regioselectivity of the cleavage process.

Additionally, the reaction exhibited solvent-dependent behavior. When conducted in solvents such as nitromethane and dichloromethane, γ-ketophosphonates were produced, but yields were generally lower compared to solvent-free conditions. This suggests that the absence of solvent may further enhance carbocation stability, allowing the reaction to proceed more efficiently.

Additionally, the study indicated that unsymmetrical 1,3-diketones preferentially form γ-ketophosphonates (93) and (94) due to the stability of their enol forms. The reaction favored enol over keto forms in these diketones, leading to a preference for cleavage at specific sites and yielding higher proportions of γ-ketophosphonates.

**Scheme 26:**
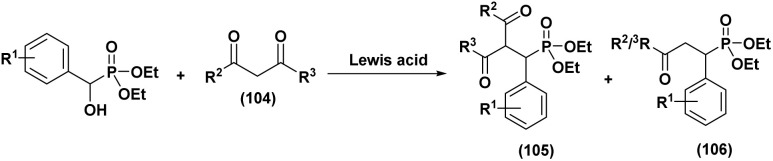
α-aryl substituted γ-ketophosphonates: Lewis acid mediated reactions of 1,3-diketones with α-hydroxyphosphonates.

The authors propose a mechanism where the Lewis acid facilitates the departure of a hydroxyl group from the α-hydroxyphosphonate, generating a carbocation intermediate. This intermediate is stabilized by the phosphoryl group, crucial for enabling the subsequent regioselective C–C bond cleavage in the 1,3-diketone. This method provides an environmentally friendly alternative to traditional syntheses of γ-ketophosphonates, avoiding toxic metal catalysts and excessive solvents (Scheme 26).

A Japanese research team^116^ has presented a practical approach for the kinetic resolution (KR) of racemic α-hydroxyphosphonates (112), leading to the formation of chiral α-acyloxyphosphonates (111) and α-hydroxyphosphonates (112). The method employs pivalic anhydride (109), a chiral acyl-transfer catalyst ((*R*)-benzo-tetramisole ((*R*)-BTM)) (110), and various carboxylic acids (108). The KR method demonstrated high enantioselectivity, with *s*-values ranging from 33 to 518, and proved versatile across different α-hydroxyphosphonate substrates.

To optimize conditions, the authors evaluated the reaction in diethyl ether and THF, with THF yielding the best results: the chiral ester (*S*) was obtained with a 46% yield and 99% enantiomeric excess (ee), while the recovered chiral alcohol (*R*) achieved 88% *ee*, corresponding to an impressive s-value of 419. The impact of different carboxylic acids on enantioselectivity was then explored, with diphenylacetic acid showing the highest *s*-value (419). Although other carboxylic acids, such as acetic, propanoic, and cyclohexanecarboxylic acids, also yielded esters with good selectivity and yields, they achieved lower s-values.

The KR was successfully applied to various α-hydroxyphosphonate substrates with different substituents (*e.g.*, Me, Et, Ph) on the phosphonate group. Substituent type significantly influenced yield and enantioselectivity; diethyl α-hydroxyphosphonate (±) exhibited high enantioselectivity (*s* = 337) with 46% yield for the ester and 52% for the recovered alcohol, whereas the bulkier diphenylphosphonate showed slightly lower enantioselectivity with an s-value of 106.

The scope of KR was expanded by applying it to dimethyl α-hydroxyphosphonates with varying alkyl and functional groups adjacent to the phosphonate. The results showed high selectivity (*s*-values up to 518) and enantiomeric excess for the recovered alcohols. However, bulky substituents (*e.g.*, *t*-bu) affected selectivity, requiring adjustments in acyl donors and reaction conditions to optimize yields and enantioselectivity.

**Scheme 27:**
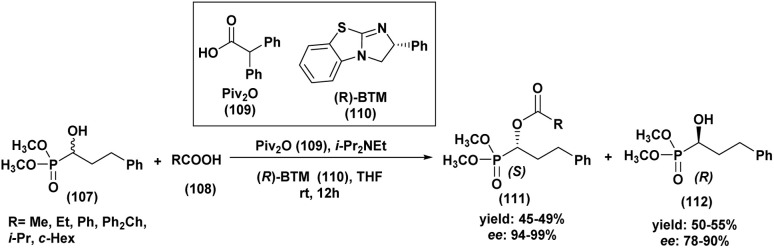
Kinetic resolution of racemic 1-hydroxy-3-phenylpropylphosphonate ((±)-1) with a variety of carboxylic acids.

DFT calculations provided insight into the transition states responsible for selective acylation. The preferred transition state for forming the (*S*)-ester showed a stabilizing interaction between the ester carbonyl oxygen and the positively charged catalyst intermediate, favoring selective acylation of (*S*)-alcohols over (*R*)-alcohols. The higher energy associated with the (*R*)-ester transition state accounts for the high enantioselectivity observed for the (*S*)-product (Scheme 27).

In the oxidation of α-hydroxyphosphonates^117^ to α-ketophosphonates (115), the study identifies the AuPd bimetallic nanoparticles (specifically with a 1 : 1 Au ratio) as particularly effective. This is significant because α-hydroxyphosphonates are challenging to oxidize directly due to the high sensitivity of C(O)–P bonds to both acidic and basic conditions, which can lead to unwanted hydrolysis or decomposition. The choice of base-free, mild conditions offered by AuPd nanoparticles avoids these issues, providing an advantage over traditional catalytic approaches that typically require harsher environments.

**Scheme 28:**
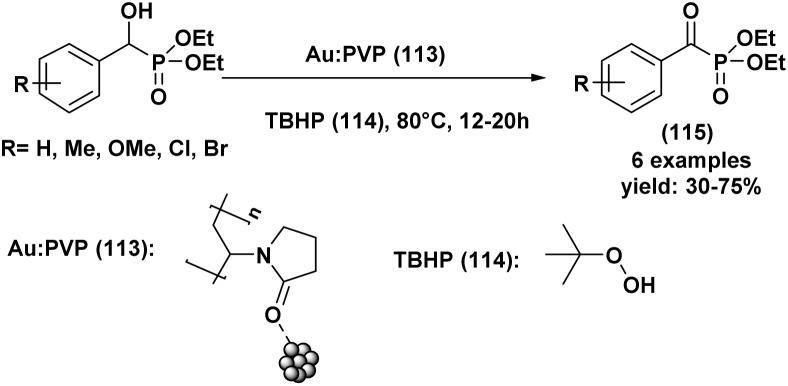
Oxidation of α-hydroxypghosphonates to α-ketophosphonates.

The authors report that using only molecular oxygen (O_2_) as the oxidant leads to partial hydrolysis, resulting in aldehyde formation rather than the desired α-ketophosphonate product (115). This side reaction emphasizes the necessity of carefully balancing oxidation conditions when working with sensitive phosphonate substrates. To address this, the addition of *tert*-butyl hydroperoxide (TBHP) (114) as a secondary oxidant alongside molecular oxygen substantially improved the selectivity for α-ketophosphonates. TBHP likely generates free radicals that are stabilized by the nanoparticle surface, promoting a reaction pathway that selectively forms the ketone without compromising the phosphonate group (Scheme 28).

The results show that the oxidation yields varied with the electronic nature of substituents on the aromatic ring of the hydroxyphosphonates. Electron-donating substituents, such as -OMe and -Me, facilitated higher product yields compared to electron-withdrawing groups like –NO_2_, which showed reduced activity. This difference suggests that electron density on the aromatic ring may influence the interaction between the substrate and the AuPd catalytic surface, as well as the efficiency of the oxidation pathway.

The authors propose that AuPd alloy nanoparticles facilitate the activation of molecular oxygen and TBHP, leading to the formation of superoxo-like species that interact with the hydroxyphosphonate substrates. The synergistic effect of Pd electron-donating capacity towards Au in the alloy enhances this activation process, stabilizing reactive intermediates essential for selective oxidation. This study demonstrates that the compositional tuning of AuPd catalysts can selectively drive oxidation reactions for sensitive compounds like hydroxyphosphonates under mild and environmentally friendly conditions, marking an advance in the catalytic transformation of phosphorus-containing compounds. Further research into the recyclability and stability of these nanocatalysts could provide additional insights for sustainable oxidation processes.

In 2018, Kerim *et al.*^118^ reported a significant advancement in the synthesis of allyl-α-hydroxyphosphonates and phosphono-oxaheterocycles (117), (120) and (122) through a novel palladium-catalyzed *O*-allylation of α-hydroxyphosphonates, followed by ring-closing metathesis (RCM). Traditionally, allylation reactions for these compounds pose challenges, primarily due to the reactivity of phosphonate groups and the tendency for competing side reactions, such as phospha-Brook rearrangements, under basic conditions. In this work, the authors achieved a mild and selective pathway by using palladium catalysis, which uniquely promotes *O*-allylation without requiring strong bases that can trigger unwanted rearrangements or decomposition of the α-hydroxyphosphonate intermediates.

The mechanistic pathway appears to rely on palladium-catalyzed allylic alkylation (Pd-AA), which successfully enables *O*-allylation over the commonly observed C-allylation or rearrangement side products. This *O*-allylation step is especially remarkable because the palladium catalyst system, combined with a stabilizing agent such as Cs_2_CO_3_, avoids decomposition and side reactions by preventing the usual phosphonate breakdown pathways. The addition of Cs_2_CO_3_ stabilizes the α-hydroxyphosphonate *in situ*, enabling effective allylation at the oxygen center rather than causing phospha-Brook rearrangements. The choice of palladium as a catalyst likely plays a role in coordinating with the allyl groups and directing the reaction toward *O*-allylation. Notably, the authors demonstrated that these conditions yield high selectivity and conversion rates, highlighting the procedure's robustness across a diverse array of substrates, including electron-rich, electron-poor, aromatic, and aliphatic α-hydroxyphosphonates.

**Scheme 29:**
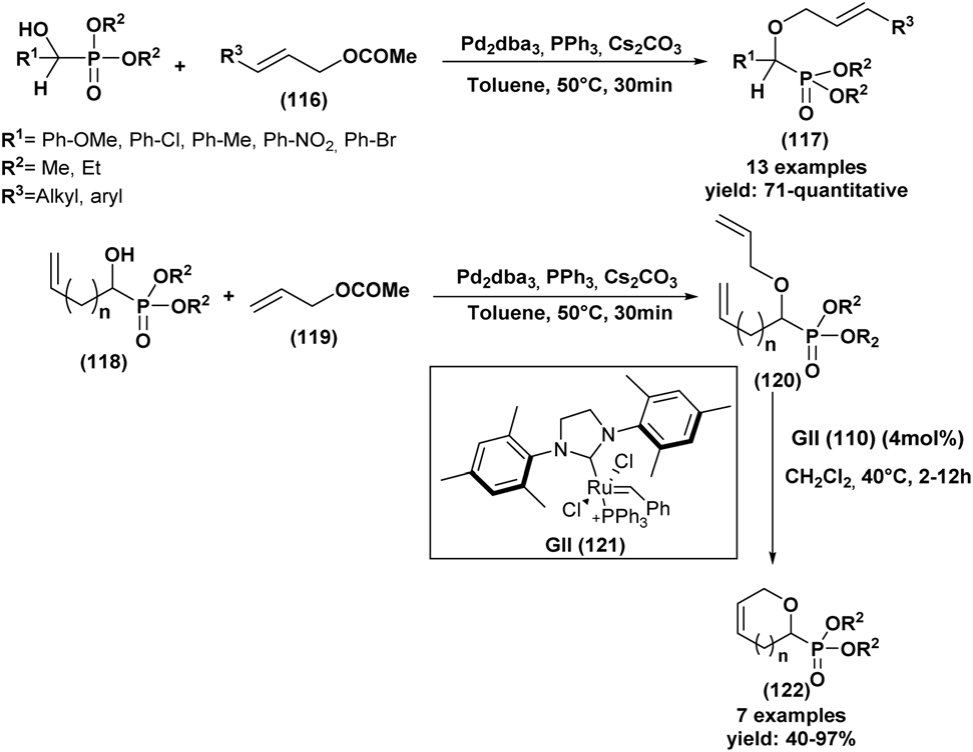
Using α-hydroxyphosphonate in the synthesis of allyl and oxa-heterocyclic phosphonate compounds.

Following *O*-allylation, the introduction of a ring-closing metathesis (RCM) step allows for the formation of phosphono-oxaheterocycles (122), which are valuable in medicinal chemistry and biomolecular applications due to their resemblance to phosphorylated sugars and natural glycosides. The RCM step proved highly efficient with small rings, yielding phosphono-oxaheterocycles of five and six members with high purity. However, for larger rings and macrocycles (12–16 membered), reaction conditions had to be adjusted by diluting the reaction concentration to prevent oligomerization, a common issue in the formation of larger rings. This adaptability to macrocycle formation demonstrates the method's versatility and suggests a strong potential for synthesizing diverse cyclic structures (Scheme 29).

**Scheme 30:**
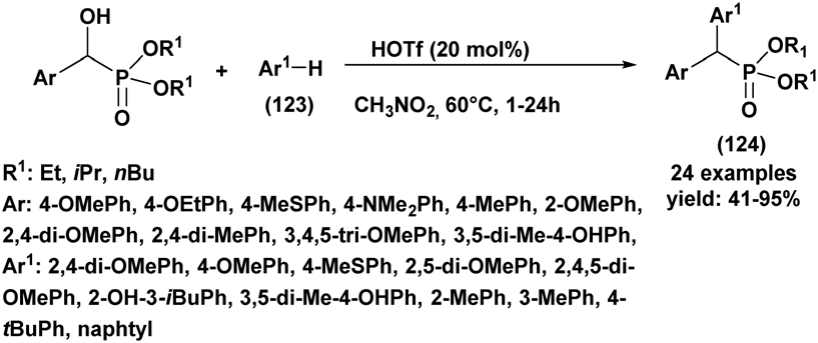
Synthesis of α-diarylmethylphosphonates by HOTf catalyzed Friedel–Crafts arylation of α-aryl α-hydroxyphosphonates.

In the same year, Chen *et al.*^119^ published a new method for synthesizing α-diarylmethylphosphonates (124) through the condensation of α-hydroxyphosphonates with hetero-aromatic compounds (123)*via* Friedel–Crafts arylation, using HOTf as an effective Brønsted acid catalyst. Although various Lewis acids were initially tested, HOTf proved to be optimal, achieving yields of up to 99% in nitromethane within just 0.5 hours. Testing of different hetero-aromatic compounds (123) showed that activated arenes, such as 1,3-dimethoxybenzene and anisole, provided high yields with excellent regioselectivity, while unactivated arenes required a higher catalyst loading and yielded some regioisomeric mixtures. The study expanded the scope of α-hydroxyphosphonates 23, finding that electron donating and certain steric substituents on the phenyl ring generally enhanced yields, although *ortho*-substitution hindered reactivity. The proposed mechanism involves a carbocationic intermediate stabilized by a *para*-methoxyphenyl group, enabling arylation despite the electron-withdrawing phosphoryl group. This metal-free, high-yielding approach with the affordable HOTf catalyst offers a promising and practical route for synthesizing α-diarylmethylphosphonates (124), with ongoing research exploring further applications and catalyst improvements (Scheme 30).

A 2019 study^120^ titled “Synthesis of organophosphates starting from α-hydroxyphosphonates” presents a method for converting α-hydroxyphosphonates into organophosphates (125) using the phospha-Brook rearrangement under phase transfer catalytic conditions. The authors tested several catalysts and found that Cs_2_CO_3_ and K_2_CO_3_ were effective for the rearrangement, with Cs_2_CO_3_ proving to be the most efficient, achieving complete conversion and high yields.

**Scheme 31:**
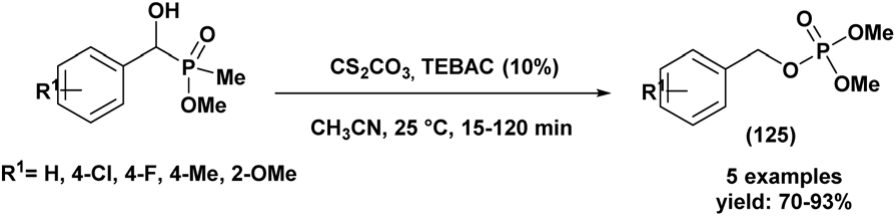
Synthesis of organophosphates starting from α-hydroxyphosphonates using Cs_2_CO_3_ and TEBAC.

Initial trials using triethylamine at room temperature did not yield any reaction. However, introducing bases like DBU facilitated some conversion, albeit with decomposition. The use of a phase transfer catalyst, TEBAC, alongside solid bases significantly improved both selectivity and yield. The study also explored the impact of substituents on reactivity, noting that electron-withdrawing groups (*e.g.*, 4-Cl, 4-F) enhanced reactivity, while electron-donating groups reduced it. Overall, the developed method provides a cleaner and more efficient pathway to organophosphates, particularly when employing Cs_2_CO_3_ and phase transfer catalysis (Scheme 31).

Our group^121^ published the synthesis of various sulfamidocarbonyloxyphosphonate derivatives (117) through a two-step process. The synthesis began with commercial chlorosulfonyl isocyanate (CSI) (114) and α-hydroxyphosphonates, which served as the base structure for the phosphonate functionality. These α-hydroxyphosphonates were either commercially available or synthesized in an earlier step.

Under anhydrous conditions, CSI reacted with α-hydroxyphosphonates at low temperatures (around 0 °C). The reaction was conducted in an anhydrous environment to prevent side reactions, particularly hydrolysis. This step produced an *N*-chlorosulfonyl carbamate intermediate (127), a crucial precursor that introduced the carbamoyl group essential to the final sulfamidocarbonyloxyphosphonate structure (129). The intermediate formed with high efficiency, typically achieving near-quantitative yields. The obtained *N*-chlorosulfonyl carbamate intermediate (127) was then reacted with various primary and secondary amines (128), allowing for the synthesis of diverse sulfamidocarbonyloxyphosphonate derivatives. This diversity was achieved by selecting different amine groups, which influenced the biological and physicochemical properties of the final compounds. The amine reaction was also conducted at low temperatures (approximately 0 °C) with triethylamine as a base to neutralize acidic by-products and drive the reaction to completion. The final sulfamidocarbonyloxyphosphonate derivatives (129) were isolated with high purity and excellent yields (92–99%) within short reaction times (60–90 minutes).

**Scheme 32:**
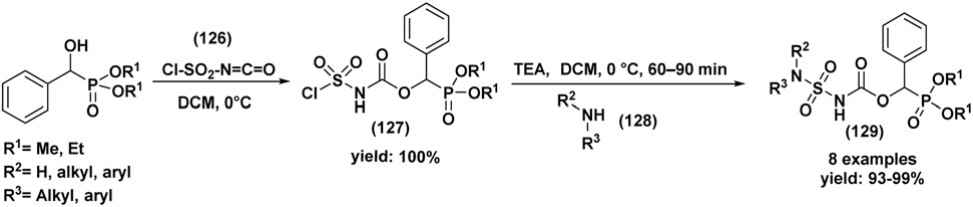
Synthesis of sulfamidocarbonyloxyphosphonates staring from α-hydrophosphonates.

To broaden the scope and variety of synthesized compounds, additional series were created using (*S*)-amino acid esters and oxazolidin-2-one. These variations resulted in compounds with excellent yields, further enhancing the structural diversity of the sulfamidocarbonyloxyphosphonate derivatives (Scheme 32).

Another paper^122^ reports the development of an efficient method for preparing optically active α-hydroxyphosphonates (132) through the stereoselective esterification of α-hydroxyphosphonates with (+)-dibenzoyl-L-tartaric anhydride (d-Bz-L-TA) (130), using Bi(OTf)_3_ as a stereoselective catalyst. The synthesis began with racemic diethyl 1-hydroxy-1-phenylmethylphosphonate, prepared according to established methods. This racemic mixture was then combined with d-Bz-L-TA (130) in a suitable solvent under optimized conditions to promote stereoselective esterification.

Various Lewis acids were tested to enhance the stereoselectivity of the reaction. Common catalysts like FeCl_3_, ZnCl_2_, AlCl_3_, and BiCl_3_ demonstrated poor selectivity or low yields. However, Bi(OTf)_3_ proved to be the most effective catalyst, offering high selectivity and eco-friendly properties. The reaction was conducted in different solvents, such as CH_2_Cl_2_, dioxane, and toluene, with CH_2_Cl_2_ yielding the best results in terms of both yield and diastereomeric ratio. Temperature variations from −40 °C to room temperature revealed that rt was optimal, balancing reaction speed and stereoselectivity.

**Scheme 33:**
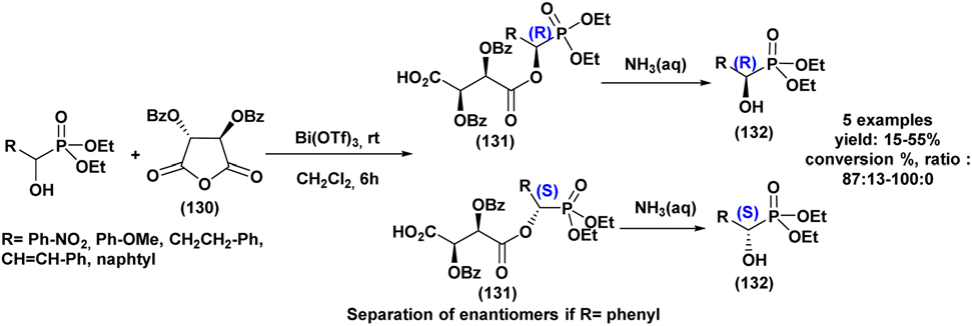
Diastereoselective reaction of α-hydroxyphosphonates with d-Bz-L-TA in the presence of Bi(OTf)_3_.

Using Bi(OTf)_3_ at 15 mol%, the reaction achieved an optimal 83 : 17 ratio of diastereomers esters (131) (*R*/*S*) with a 58% yield over 24 hours. After the reaction, the diastereomeric mixture was separated by column chromatography. The major diastereomer, 131 (*R*), was isolated in pure form through column chromatography, followed by hydrolysis to yield enantiomerically pure (*R*)-1-hydroxy-1-phenylmethylphosphonate 132 (*R*) (Scheme 33).

Keglevich and Rádai^123^ investigated the reversibility of α-hydroxyphosphonates formation in the Kabachnik–Fields reaction for α-aminophosphonate derivatives, confirming this through rearrangement and trapping experiments. In their study, dimethyl α-hydroxybenzylphosphonate was treated under phase-transfer catalytic conditions with an alkali carbonate base in acetone, resulting in its rearrangement to benzylphosphate. Additionally, the formation of dimethyl α-hydroxyisopropylphosphonate was observed, demonstrating that α-hydroxyphosphonate can reversibly decompose and reform under these conditions.

Using different bases (Cs_2_CO_3_ and K_2_CO_3_), the reaction displayed varying rates of conversion, with Cs_2_CO_3_ enabling the fastest transformation. Reaction progress and product distribution were monitored over time, showing that longer reaction times or higher temperatures led to increased yields of benzylphosphate. These findings confirm the reversible nature of α-hydroxyphosphonate formation in the process of aminophosphonate (134) synthesis *via* an amine (133) condensation. This reversibility implies that α-hydroxyphosphonates can decompose back into their starting materials, which can then recombine in alternative configurations, supporting the concept of reversible intermediates in this reaction.

**Scheme 34:**
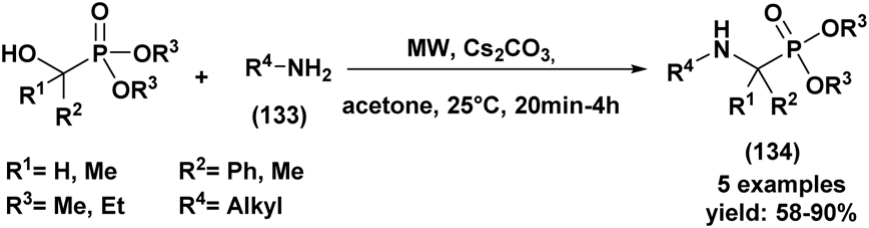
Preparation of α-aminophosphonates from condensation of α-hydroxyphophonates with primary amine.

The results are significant as they provide valuable insights into the mechanistic pathways of the Kabachnik–Fields reaction and open up opportunities for further manipulation of these intermediates in synthetic applications (Scheme 34).

**Scheme 35:**
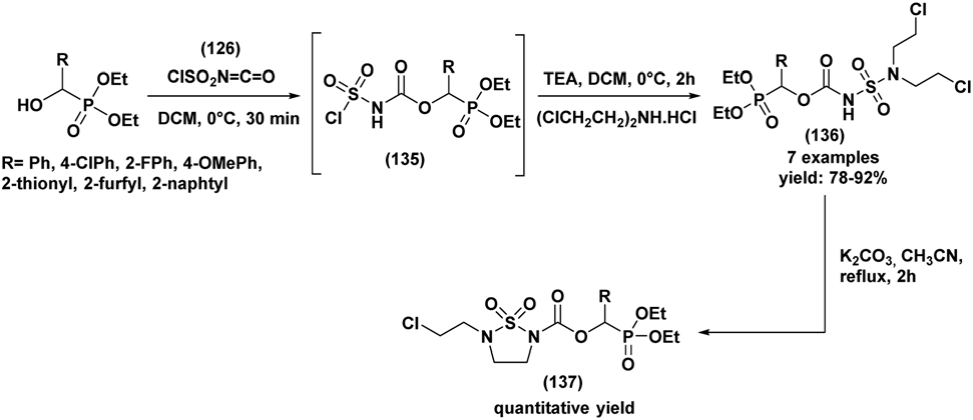
Synthesis of cyclic *N*-2-chloroethylsulfamide starting from α-hydroxyphosphonates.

In 2021, our group^124^ reported the synthesis of novel 5-(2-chloroethyl)-1,2,5-thiadiazolidine-2-carbaldehyde-1,1-dioxide compounds (137) with a phosphonate moiety and evaluated their antimicrobial properties, starting from hydroxyphosphonate derivatives. The synthesis followed a multi-step pathway, beginning with the formation of α-hydroxyphosphonates *via* the Abramov reaction of dialkylphosphite with aromatic aldehydes under solvent-free conditions, achieving high yields (88% to 97%) within 10 minutes. Subsequently, the α-hydroxyphosphonates were treated with CSI (126) in anhydrous DCM at 0 °C to introduce a chlorosulfonyl carbamoyl group, yielding *N*-chlorosulfonyl carbamate intermediates (135) in 30 minutes. These intermediates were then reacted with bis(2-chloroethyl)amine hydrochloride in DCM, with triethylamine as a base at 0 °C, producing sulfamidocarbonyloxymethylphosphonates (136) in good yields (78–92%) after stirring for 2 hours. Finally, this compounds underwent cyclization using K_2_CO_3_ as a base in refluxing acetonitrile, promoting intramolecular cyclization and resulting in the final cyclic products (137) in quantitative yields (Scheme 35).

## 5. Conclusion

In summary, this review highlights advancements in the synthesis, reactivity, and biological applications of α-hydroxyphosphonates from 2010 to 2025. Classical methods such as the Pudovik and Abramov reactions continue to dominate, while growing interest in alternative synthetic approaches including microwave-assisted, ultrasonication, and catalytic protocols has opened more sustainable and efficient pathways. The development of asymmetric synthesis, employing chiral organocatalysts and metal-based systems, has further enabled access to enantioenriched derivatives with high functional value. In addition to classical approaches, alternative synthetic methods such as aldol condensations, Michaelis–Arbuzov reactions, oxidation, and catalytic hydrogenation have emerged as valuable tools for accessing α-hydroxyphosphonates. These routes offer enhanced stereocontrol and broaden the structural diversity of this class, supporting their use in designing bioactive molecules.

The reactivity of the α-hydroxyphosphonate scaffold, particularly through its free hydroxyl group, provides a platform for diverse transformations leading to structurally elaborate compounds. This includes α-substituted variants such as amino, keto, and halogenated phosphonates, which expand their utility in both chemical synthesis and drug design.

Biologically, α-hydroxyphosphonates demonstrate a wide array of pharmacological activities, ranging from antiviral and antibacterial to anticancer and antioxidant effects. Their resemblance to α-hydroxycarboxylic acids contributes to their bioisosteric relevance and therapeutic potential. Complementary computational studies, including DFT and molecular docking, have offered mechanistic insights and supported structure activity relationship modeling.

This review aims to serve as a reference for researchers by summarizing recent advances in α-hydroxyphosphonate chemistry, supporting future developments in synthesis, reactivity, and bioactive applications.

## Author contributions

Zineb Aouf coordinated the project and supervised the overall structure of the manuscript. Abdeslem Bouzina, Djawhara Chohra, Rihab Benabes, Yousra Ouafa Bouone, Rayene Sayad, and Houria Bentoumi participated in literature collection, analysis, and drafting of specific sections. Malika Ibrahim-Ouali and Nour-Eddine Aouf provided critical revisions and scientific guidance. All authors contributed to the writing, reviewed the manuscript thoroughly, and approved the final version for submission.

## Conflicts of interest

The authors report no conflicts of interest related to this work.

## Data Availability

This study did not involve the generation or analysis of any datasets.
